# Regulated N-glycosylation in the endoplasmic reticulum controls chaperone function and receptor trafficking

**DOI:** 10.1126/science.adp7201

**Published:** 2024-11-07

**Authors:** Mengxiao Ma, Ramin Dubey, Annie Jen, Ganesh V. Pusapati, Bharti Singal, Evgenia Shishkova, Katherine A. Overmyer, Valérie Cormier-Daire, Juliette Fedry, L. Aravind, Joshua J. Coon, Rajat Rohatgi

**Affiliations:** 1 Departments of Biochemistry and Medicine, Stanford University School of Medicine, Stanford, CA 94305, USA; 2 National Center for Quantitative Biology of Complex Systems, Madison, WI 53706, USA; 3 Morgridge Institute for Research, Madison, WI 53515, USA; 4 Department of Biomolecular Chemistry, University of Wisconsin, Madison, WI 53506, USA; 5 Department of Chemistry, University of Wisconsin, Madison, WI 53506, USA; 6 Université de Paris Cité, Génétique clinique, INSERM UMR 1163, Institut Imagine, Hôpital Necker-Enfants Malades (AP-HP), Paris, France; 7 National Center for Biotechnology Information, National Library of Medicine, National Institutes of Health, Bethesda, MD 20894, USA; 8 MRC Laboratory of Molecular Biology, Cambridge, CB2 0QH, UK; 9 Stanford SLAC CryoEM Initiative, Stanford, CA 94305, USA

## Abstract

One-fifth of human proteins are N-glycosylated in the endoplasmic reticulum (ER) by two oligosaccharyltransferases, OST-A and OST-B. Contrary to the prevailing view of N-glycosylation as a housekeeping function, we identified an ER pathway that modulates the activity of OST-A. Genetic analyses linked OST-A to HSP90B1, an ER chaperone for membrane receptors, and CCCD134, an ER protein we identify as the first specificity factor for N-glycosylation. During its translocation into the ER, a N-terminal peptide in HSP90B1 functions as a pseudosubstrate inhibitor of OST-A and templates the assembly of a specialized ER translocon complex containing CCDC134. Unexpectedly, OST-A functions as a scaffold rather than an enzyme in this context, stabilizing HSP90B1 by preventing its hyperglycosylation and degradation. Disruption of this pathway impairs WNT signaling at the cell surface and causes the bone developmental disorder Osteogenesis Imperfecta. Thus, N-glycosylation can be regulated by ER factors to control cell-surface receptor signaling and tissue development.

## Introduction

Asparagine-linked glycosylation or N-glycosylation in the endoplasmic reticulum (ER) is a pervasive post-translational modification in eukaryotes found on ~20% of the proteome (1, 2). N-glycosylation influences myriad processes, including tissue development, cell-cell recognition in the immune system, inflammatory responses and the metastatic spread of cancer cells (3, 4).

The dominant view in cell biology has been that N-glycosylation is a constitutive process initiated by two multi-subunit oligosaccharyltransferase complexes, OST-A and OST-B, that contain the distinct catalytic subunits STT3A and STT3B, respectively (5). OST-A, which contains unique subunits (OSTC, KRTCAP2) that anchor it to the SEC61 protein channel (6), functions co-translationally to transfer a pre-assembled glycan from a lipid donor to asparagine residues found in the context of N-X-S/T/C sequons (where X can be any residue other than proline). Structural studies show that the catalytic site in STT3A is positioned at the luminal surface of the ER membrane to scan the nascent chain for sequons as it emerges from SEC61 (7–10). OST-B functions co- or post-translationally on sequons missed or poorly recognized by OST-A (5).

An important unanswered question is whether the activity of the OST-A and OST-B complexes, which sit at the apex of the N-glycosylation pathway, can be controlled by other factors to switch the glycan occupancy of specific sequons on or off. In a screen for new components of the WNT signaling pathway, we serendipitously discovered the first example of a signaling module that controls the activity of OST-A. This pathway regulates the sensitivity of cells to diverse extracellular signals received at the cell surface, providing a mechanism for communication between the ER and the plasma membrane.

### Genetic screens identify ER-localized regulators of WNT signaling

**Figure 1:**
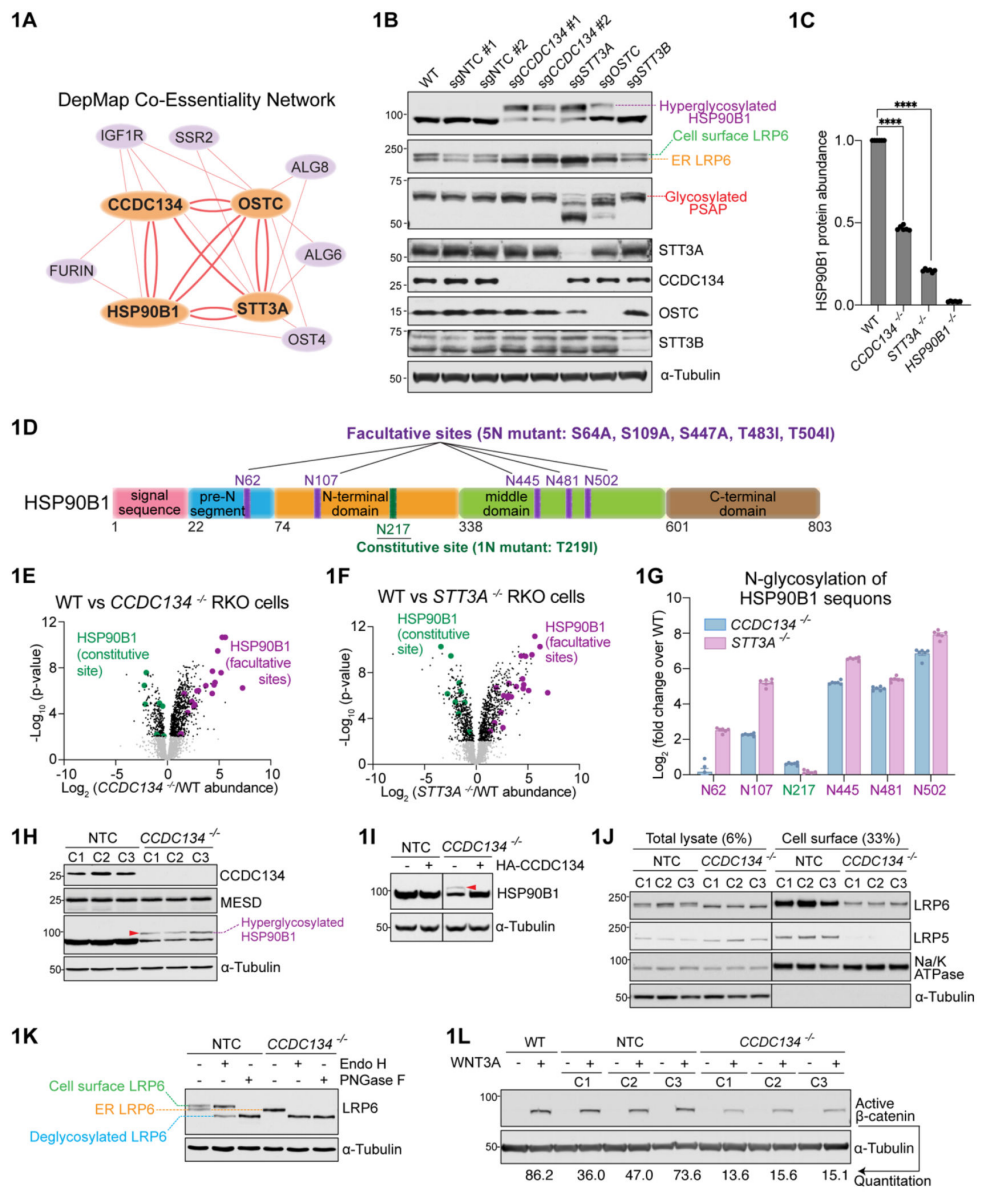
Regulation of HSP90B1 N-glycosylation and WNT signaling by an ER protein network. **(A)** DepMap co-essentiality relationships between CCDC134, STT3A, HSP90B1, and OSTC visualized using *Fireworks* (16). The bi-directional edges between these four genes (bold orange lines) indicate concordant effects on cell growth across >1000 cell lines. **(B)** Abundances and glycosylation status of HSP90B1, its client LRP6, and PSAP (an exclusive OST-A substrate) in lysates from cells expressing a control (NTC) sgRNA or sgRNA’s against the indicated genes. **(C)** HSP90B1 protein abundance in wild-type (WT), *CCDC134*^*-/-*^, *STT3A*^*-/-*^, and *HSP90B1*^*-/-*^ RKO cell lines measured by mass spectrometry, normalized to WT cells. Bars represent the mean abundance +/- SEM from six independent mass spectrometry runs, represented as individual data points. Statistical significance was determined by one-way ANOVA with Dunnett’s multiple comparisons test; **** p<0.0001. **(D)** Domain architecture of HSP90B1. The constitutive and facultative N-glycosylation sites are labeled in green and purple, respectively. The point mutations used to disrupt the one constitutive sequon (1N mutant) and the five facultative sequons (5N mutant) are listed. fig.S2A shows these features on the HSP90B1 three dimensional structure using the same coloring scheme. **(E, F)** Abundances of glycosylated peptides in *CCDC134*^*-/-*^ and *STT3A*^*-/-*^ cells measured using global, unbiased N-glycoprotemics. Each data point represents the fold change in abundance of a distinct glycopeptide (defined by sequence and glycan structure) in mutant compared to wild-type cells. Glycopeptides that include the constitutive and facultative sequons in HSP90B1 are colored green and purple, respectively. Full dataset in Data S2. **(G)** Enrichment of glycopeptides (normalized to total HSP90B1 protein abundance) that include each of the facultative and constitutive sequons of HSP90B1 in *CCDC134*^*-/-*^ or *STT3A*^*-/-*^ cells compared to WT cells. Bars show the mean +/- SEM from six independent mass spectrometry runs, represented as individual data points. The abundances of all peptides that include each HSP90B1 sequon were integrated using individual peptide data summarized in **1E and 1F** and provided in Data S2. **(H, I)** Abundance and glycosylation status of HSP90B1 in lysates from three (C1-C3) independent control (NTC) and *CCDC134*^*-/-*^ clonal cell lines (**H**), or after the stable expression of HA-tagged CCDC134 in *CCDC134*^*-/-*^ cells (**I**). The hyperglycosylated form of HSP90B1 is denoted by a red arrowhead in this and all subsequent panels. **(J)** LRP6 and LRP5 abundances in total lysate or at the plasma membrane from three independently derived (C1-C3) control (NTC) or *CCDC134*^*-/-*^ clonal cell lines. The cell surface protein Na/K ATPase and cytoplasmic protein α-tubulin serve as controls, both for loading and for the specificity of cell surface biotinylation. **(K)** Glycosidase sensitivity in conjunction with mobility on SDS-PAGE gels was used to measure the ER or cell-surface pools of LRP6 in control or *CCDC134*^*-/-*^ cells. Endoglycosidase H (Endo H) can remove glycans added in the ER but not the complex glycan modifications added in the Golgi; Peptide-*N*-Glycosidase F (PNGase F) can remove glycans on both ER and cell-surface proteins. **(L)** Active (non-phosphorylated) β-catenin abundance (a metric of WNT signaling strength) was measured (+/- WNT3A) using immunoblots in wild-type cells or clonal cell lines expressing a control (NTC) sgRNA or an sgRNA targeting *CCDC134*.

Using a fluorescence-based transcriptional reporter, we conducted a genome-wide, loss-of-function CRISPR/Cas9 screen in a human cell line (RKO) to identify genes required to activate WNT/β-catenin signaling in response to WNT3A (fig.S1A and Data S1) (11). The screen identified many known WNT signaling components (fig.S1B and S1C): β-catenin, LRP6 (a co-receptor for WNT ligands), and two ER chaperones, MESD and HSP90B1, that facilitate LRP5 and LRP6 (LRP5/6) folding in the ER (12, 13). Our screens also identified three genes not clearly linked to WNT signaling: *CCDC134, STT3A* and *OSTC* (fig.S1B, S1C, and S1D). STT3A and OSTC are two subunits of the OST-A (but not the OST-B) complex. CCDC134 is a poorly studied protein required for mouse embryonic development (14). CCDC134, STT3A and OSTC showed a strong signature of genetic co-essentiality with each other and the LRP6 chaperone HSP90B1 in the Cancer Dependency Map (DepMap) (Fig.1A) (15, 16).

While STT3A, OSTC and HSP90B1 are established ER proteins, CCDC134 is annotated as either a secreted cytokine or a nuclear protein in Uniprot (17, 18). However, CCDC134 contains signal sequences for both ER targeting and retention (fig.S1E). Experiments confirmed that CCDC134 is a protein resident in the ER: mutation of its C-terminal ‘QSEL’ ER retention sequence to ‘QSSA’ abrogated its ER retention and enabled its secretion into the media (fig.S1F and S1G).

### Regulation of the ER chaperone HSP90B1 by hyperglycosylation

A review of the literature revealed a connection between STT3A and HSP90B1. Disruption of *STT3A* (but not *STT3B*) results in hyperglycosylation and de-stabilization of HSP90B1 (19). We reproduced this observation and, to our surprise, found that disruption of *CCDC134* and *OSTC* also resulted in the hyperglycosylation and destabilization of HSP90B1, supporting DepMap’s prediction of a shared function amongst these proteins (Fig.1B and 1C). Additionally, the loss of STT3A, CCDC134 or OSTC reduced the abundance of the WNT co-receptor LRP6 at the cell surface (Fig.1B), explaining why loss of these proteins impaired responses to WNT ligands in our screen (fig.S1C and S1D).

The function and regulation of HSP90B1 hyperglycosylation has remained mysterious for three decades (19–23). HSP90B1 has six glycosylation sites conserved in evolution (Fig.1D and fig.S2A). One of these sequons (N217) is constitutively modified with an N-glycan (hereafter called the “constitutive sequon”) while five (N62, N107, N445, N481 and N502) are only used under specific conditions (hereafter called “facultative sequons”) (19, 20, 22). HSP90B1 hyperglycosylation has been observed when its abundance increases, either when it is overexpressed or when its transcription increases in response to ER stress (20).

Facultative sequons are located in regions of HSP90B1 that would be predicted to impair protein function or folding (20) (fig.S2A). This arrangement is unusual: in most secretory pathway proteins, sequons have been depleted during evolution from buried regions of proteins (24). Hyperglycosylated HSP90B1 cannot fold into a functional protein, causing it to be flagged and degraded by the ERAD machinery (fig.S2B).

Global N-glycoprotemics revealed both highly specific and concordant effects of STT3A and CCDC134 on the N-glycosylation of HSP90B1 (25). Out of ~4000 N-glycopeptides identified across the proteome, those derived from HSP90B1 showed the greatest increase in abundance in *CCDC134*^-/-^ and *STT3A*^*-/-*^ cells compared to wild-type cells (Fig.1E and 1F, Data S2), even though the overall abundance of HSP90B1 was reduced in both mutant cell lines (Fig.1C). In both mutant cell lines, these N-glycopeptides encompassed one of the five facultative sequons of HSP90B1 (Fig.1G). In contrast, the abundance of N-glycopeptides containing the single constitutive sequon (N217) did not change in *CCDC134*^-/-^ and *STT3A*^*-/-*^ cells compared to wild-type cells (Fig.1G)

### Regulation of WNT reception at the cell surface by CCDC134 in the ER

Since the function of CCDC134 was unknown, we investigated its role in WNT signaling in more detail (26). Disruption of *CCDC134* in multiple mouse and human cell lines resulted in HSP90B1 hyperglycosylation and destabilization (Fig.1H, fig.S3A-S3F). Rescue experiments demonstrated that stable re-expression of CCDC134 at near-endogenous levels using a doxycycline-inducible system (fig.S3G) suppressed both HSP90B1 hyperglycosylation and restored its abundance (Fig.1I). In addition, the co-expression of CCDC134 suppressed HSP90B1 hyperglycosylation caused by its overexpression (fig.S3H) and had a dose-dependent protective effect on HSP90B1 in the face of ER stress (fig.S3I).

Cell-surface LRP5/6 abundances were markedly reduced in *CCDC134*^-/-^ cells (Fig.1J). Electrophoretic mobility and glycosylation analyses (27) demonstrated that LRP6 was trapped in the ER (Fig.1K). As a consequence, *CCDC134*^-/-^ cells were much less responsive to WNT ligands than wild-type cells, measured by WNT reporter activity (fig.S1C) or β-catenin protein accumulation (Fig.1L). WNT signaling sensitivity could be restored by re-expressing wild-type CCDC134, but not mutants that lack either the signal sequence for its ER targeting or the QSEL sequence for its ER retention (fig.S3J and S3K). We conclude that CCDC134 regulates WNT signaling in the ER by controlling the trafficking of LRP5/6, obligate co-receptors for WNT ligands, to the cell surface.

To establish organism-level functional and disease relevance, we used primary fibroblasts from two human patients carrying loss-of-function mutations in CCDC134 (28). CCDC134 mutations have been recently identified as a rare cause of the bone developmental disorder Osteogenesis Imperfecta (OI) in five patients from three independent families (28–30). These patients suffer from a severe, deforming subtype of OI (Type III) that is also seen in patients carrying mutations in other WNT pathway genes such as *MESDC2* and *WNT1* (31). Primary fibroblasts from *CCDC134*^-/-^ patients demonstrated HSP90B1 hyperglycosylation, reduced cell-surface LRP6 abundance and impaired WNT signaling, all defects that were reversed upon the re-expression of CCDC134 (Fig.S4).

### Genetic interactions organize CCDC134, OST-A and HSP90B1 into a pathway

**Figure 2:**
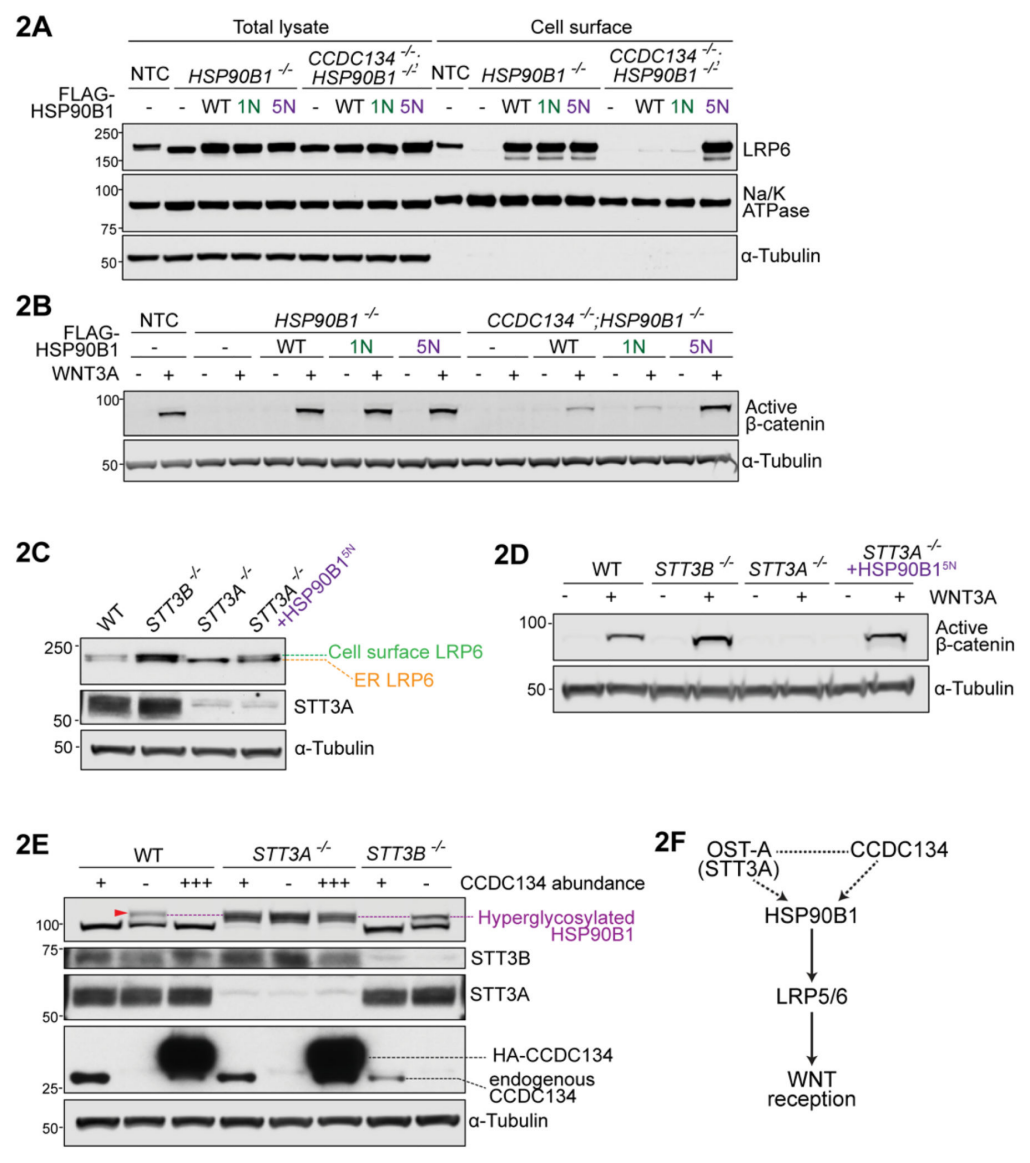
Hyperglycosylation of HSP90B1 regulates WNT signaling strength. **(A-D)** Abundances of cell-surface LRP6 (**A**,**C**) or active β-catenin abundance (**B**,**D**) in clonally derived cell lines of the indicated genotypes stably expressing wild-type (WT) FLAG-HSP90B1 or variants carrying mutations in the one constitutive (1N) or all five facultative (5N) sites (see Fig.1D). HSP90B1 variants were expressed at comparable levels (fig.S5A). **(E)** N-glycosylation status of HSP90B1 in *STT3B*^*-/-*^ and *STT3A*^*-/-*^ cells expressing different levels of CCDC134: No CCDC134 (**-**), endogenous CCDC134(**+**), stably overexpressed 3xHA-CCDC134 (**+++**) on top of endogenous CCDC134. **(F)** A provisional pathway diagram constructed based on genetic interactions (dotted lines) uncovered in our work and physical interactions (solid lines) described in the literature.

To test whether hyperglycosylation of HSP90B1 was the root cause of reduced cell-surface LRP6 and WNT sensitivity in *CCDC134*^-/-^ cells, we disrupted all five facultative sequons in HSP90B1 (hereafter called the HSP90B1^5N^ mutant) (Fig.1D). As a control, we also separately disrupted the single constitutive sequon (N217, mutated in HSP90B1^1N^). Both HSP90B1^5N^ and HSP90B1^1N^ were functional proteins because they were expressed at levels comparable to wild-type HSP90B1 (fig.S5A) and could rescue cell surface LRP6 and WNT signaling in *HSP90B1*^-/-^ cells (Fig.2A and 2B). However, only HSP90B1^5N^ (but not wild-type HSP90B1 or HSP90B1^1N^) rescued *HSP90B1*^-/-^;*CCDC134*^-/-^ double null cells, formally showing that HSP90B1 is epistatic to CCDC134 in the WNT pathway.

Analogous to the loss of either CCDC134 or HSP90B1, disruption of *STT3A* (but not *STT3B*) also markedly reduced cell-surface LRP6 and the strength of WNT signaling (Fig.2C and 2D). Remarkably, HSP90B1^5N^ expression was sufficient to completely rescue WNT signaling in *STT3A*^-/-^ cells (Fig.2C and 2D), despite the fact that the N-glycosylation of hundreds of membrane and secreted proteins is altered when OST-A function is lost (19).

There was a notable difference in HSP90B1 N-glycosylation in *STT3A*^*-/-*^ compared to *CCDC134*^*-/-*^ cells. The entire pool of HSP90B1 was hyperglycosylated in the former while only a fraction was hyperglycosylated in the latter (Fig.2E). Even a massive increase in CCDC134 abundance was unable to suppress HSP90B1 hyperglycosylation in *STT3A*^-/-^ cells (Fig.2E). These data suggest that OST-B (the only OST complex in *STT3A*^*-/-*^ cells) fully N-glycosylates HSP90B1 by default at all sequons and cannot be regulated by CCDC134. In *STT3B*^-/-^ cells, loss of CCDC134 leads to HSP90B1 hyperglycosylation--establishing that CCDC134 can regulate OST-A (Fig.2E). In the absence of CCDC134, OST-A was less efficient at N-glycosylating HSP90B1 compared to OST-B (Fig.2E), an observation we return to later.

These epistasis relationships allow the assembly of *CCDC134, HSP90B1* and *STT3A* into a provisional genetic pathway (Fig.2F) that can communicate the integrity of N-glycosylation to reception of WNT signals at the cell surface. This pathway model served as a framework for our subsequent biochemical studies.

### An N-terminal unstructured peptide in HSP90B1 regulates its own N-glycosylation

**Figure 3:**
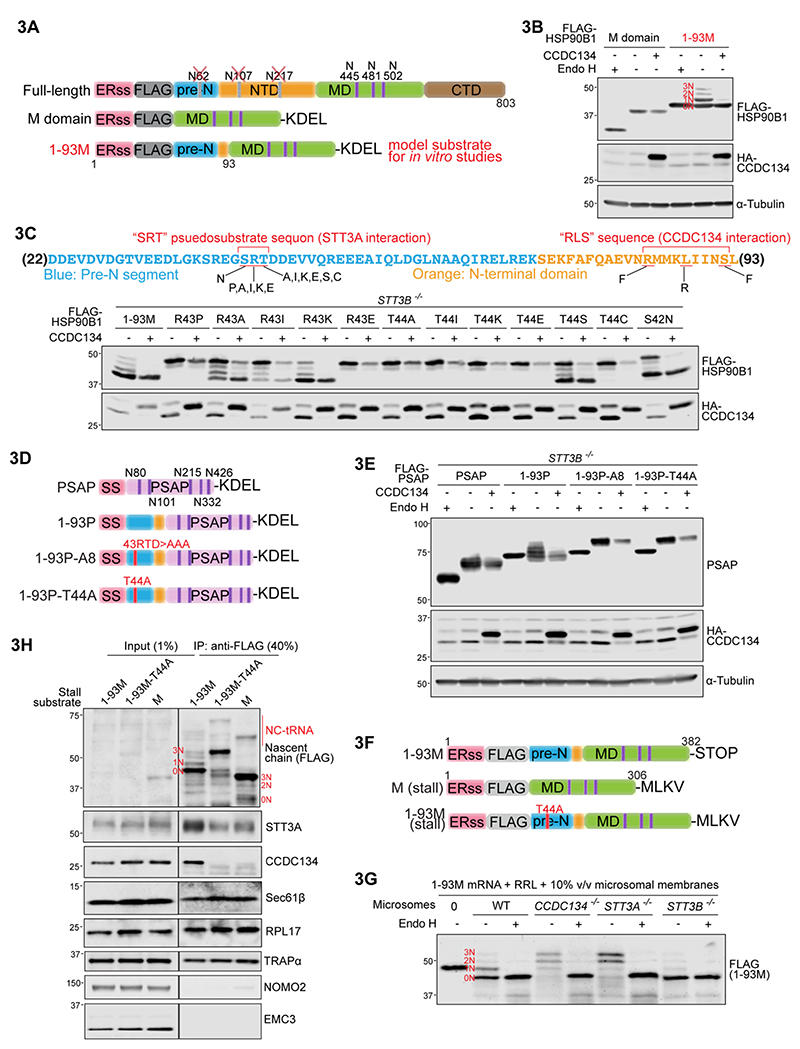
The pre-N segment of HSP90B1 inhibits its own N-glycosylation by recruiting CCDC134 to an OST-A containing secretory translocon. **(A)** Variants of HSP90B1 used for cell-based and *in vitro* assays. Key features include the ERss, ER signal sequence; FLAG, 3xFLAG tag; pre-N, unstructured segment; NTD, N-terminal domain; MD, middle domain; CTD, C-terminal domain. N-glycosylation sites in pre-N and NTD were eliminated to allow easy assessment of the glycan modification of the three sequons in the M domain by gel shifts (see **3B** and text). **(B)** Glycosylation status of HSP90B1 variants shown in **3A** was assessed using gel shifts and Endo H sensitivity after transient co-expression in HEK293T cells with WT CCDC134 (**+**) or a non-functional variant (**-**) lacking its ER signal sequence (see Fig.S3J). The four predicted N-glycoforms (carrying 0, 1, 2 or 3 glycans) are labeled 0N-3N in red lettering. **(C)** Glycosylation status of the 1-93M variant (see **3A**) of HSP90B1 carrying the indicated mutations in the “SRT” pseudosubstrate motif found in the pre-N segment. Each construct was co-expressed with functional (**+**) or non-functional (**-**) CCDC134. See fig.S6C and S6D for deletion analysis and alanine scanning mutagenesis of the pre-N segment. **(D**,**E)** Glycosylation status of chimeric proteins (**3D**) constructed by fusing variants of the 1-93 segment of HSP90B1 to the obligate OST-A substrate PSAP, which contains five N-glycosylation sites shown in **3D**. T44A changes “SRT” to “SRA” and A8 changes “RTD” to “AAA” (see **3C** and fig.S6D). All chimeras carry the ERss of HSP90B1. **(F)** Constructs used for *in vitro* translation experiments. To stably stall translation, the STOP codon was removed and a MLKV peptide sequence appended at the C-terminus. **(G)** Glycosylation status of the 1-93M variant of HSP90B1 (see **3A**) translated in rabbit reticulocyte lysate (RRL) in the presence of rough microsomal membranes generated from wild-type or clonally-derived *CCDC134*^*-/-*^, *STT3A*^*-/-*^, or *STT3B*^*-/-*^ HEK293T cells and concentrated by immunoprecipitation on anti-FLAG beads. The four N-glycoforms of 1-93M are labeled (compare to **3B**) and show sensitivity to Endo H treatment. **(H)** Association of endogenous CCDC134 with stalled 1-93M, M domain alone or a 1-93M variant carrying a T44A mutation in the “SRT” pseudosubstrate site (see **3C**). The stalled nascent chain, immunoprecipitated (IP) using anti-FLAG beads, associates with the ribosome (RPL17) and known components of the secretory translocon (STT3A, the SEC61 channel, and the TRAP complex), but not with components of the multi-pass translocon (NOMO2) or the ER-Membrane Protein Complex (EMC3) (34, 41). NC-tRNA: nascent chain-tRNA conjugates.

We next turned to investigate the question of which sequence elements within HSP90B1 regulate its own N-glycosylation. To simplify glycosylation analysis by gel shift analysis, we used a HSP90B1 variant carrying only three facultative sequons located in its middle (M) domain (Fig.3A). The M domain alone was efficiently hyperglycosylated but completely resistant to CCDC134 co-expression (Fig.3B), showing that sequences distant from the sequons themselves were required. The C-terminal domain (CTD) was dispensable, thereby implicating the N-terminal domain (NTD) and the pre-N segment (fig.S6A and S6B).

Systematic deletion analysis showed that amino acids 1-93 of HSP90B1, which includes the ER signal sequence and all of the pre-N segment, could affect the N-glycosylation of distant sequons in the M domain (fig.S6C). Deletions that extended into this 1-93 segment markedly increased the extent of M domain hyperglycosylation. A minimal model substrate, in which the unstructured N-terminal 93 amino acids of HSP90B1 were fused to its M domain carrying three facultative sequons, was used for many of our subsequent experiments (hereafter called 1-93M, Fig.3A). The hyperglycosylation of 1-93M was efficiently suppressed by CCDC134 and its shorter length enabled efficient translation and glycosylation analysis by gel electrophoresis (Fig.3B).

Alanine scanning mutagenesis of the pre-N segment identified an 18 amino acid stretch following the signal sequence that was important for the suppression of 1-93M hyperglycosylation (fig.S6D). This critical region contains a potential pseudosubstrate sequon (serine-arginine-threonine or “SRT” instead of asparagine-arginine-threonine or “NRT”) that might bind and inhibit the catalytic activity of STT3A (Fig.3C). This SRT sequence followed many of the rules of classical sequons. Alteration of the middle R to a proline (P) abolished its ability to inhibit hyperglycosylation. At the third T position, a single conservative change to alanine (hereafter “T44A”) resulted in complete N-glycosylation of 1-93M (Fig.3C) or of full-length HSP90B1, both at native facultative sequons and at ectopically introduced artificial sequons (fig.S6E). The HSP90B1-T44A mutants were also resistant to CCDC134 regulation, reminiscent of HSP90B1 in *STT3A*^*-/-*^ cells (Fig.2E). Notably, these experiments were conducted in *STT3B*^*-/-*^ cells, showing that the T44A mutation abolishes regulation of HSP90B1 N-glycosylation by both OST-A and CCDC134.

As a final test of the model that the N-terminal 1-93 segment of HSP90B1 functions as a pseudosubstrate inhibitor of the OST-A complex, we transplanted this sequence to PSAP, a protein that carries five sequons glycosylated exclusively by OST-A (Fig.3D) (5, 32). The 1-93 segment reduced the efficiency of PSAP N-glycosylation (Fig.3E), just as it reduced the efficiency of M domain N-glycosylation (Fig.3B). Secondly, it conferred sensitivity to CCDC134 in a manner that was dependent on the integrity of the SRT pseudosubstrate site (Fig.3E). We conclude that the N-terminal ~93 amino acids of HSP90B1, predicted to be largely unstructured, has two autonomous, transferable properties: it impairs the N-glycosylation of sequons that follow it in the same polypeptide and also confers sensitivity to CCDC134 (which further suppresses its N-glycosylation).

The observation that a peptide segment can regulate the N-glycosylation of distant sequons that follow it suggested a substrate-directed auto-inhibitory model: the N-terminal 1-93 sequence, which emerges from SEC61 into the ER lumen before the M domain, inhibits the ability of OST-A to N-glycosylate any following sequons in the same polypeptide. Co-translational regulation is an implicit feature of this model supported by several observations. First, regulation of HSP90B1 N-glycosylation depends on OST-A (the major co-translational oligosaccharyltransferase) but cannot use OST-B (which functions post-translationally): HSP90B1 is fully hyperglycosylated (and resistant to CCDC134) in *STT3A*^*-/-*^ cells (Fig.2E). Second, depletion of OSTC, which anchors the OST-A complex to the SEC61 channel but is not required for its integrity or catalytic activity (6), results in HSP90B1 hyperglycosylation (Fig.1B). Third, expansion of the DepMap network around the CCDC134-STT3A cluster reveals a signature of the ER translocon, including multiple subunits of the TRAP complex (fig.S7A). Finally, pulse-chase analysis was consistent with co-translational hyperglycosylation of HSP90B1 in *CCDC134*^*-/-*^ cells (fig.S7B and S7C). These observations led us to consider the model that the hyperglycosylation of HSP90B1 is regulated by a complex between its own N-terminus, CCDC134 and OST-A that assembles during its translation and translocation into the ER.

### Co-translational recruitment of CCDC134 to the ER translocon by STT3A and the HSP90B1 nascent chain

Given that CCDC134 activity depends on both STT3A (Fig.2E) and the pseudosubstrate site in HSP90B1 (Fig.3C), we sought separation-of-function mutations to test the model that CCDC134 engages both components. Potentially disruptive mutations in two ER luminal loops in STT3A (fig.S7D and S7E) increased hyperglycosylation of HSP90B1, mimicking the effect of CCDC134 loss (S7F). These loop mutations did not disrupt the integrity of the OST-A complex since the N-glycosylation of PSAP was maintained (fig.S7F). Distinct from a complete loss of STT3A (Fig.2E), HSP90B1 was only partially hyperglycosylated in these STT3A loop mutants (as it is in *CCDC134*^*-/-*^ cells), presumably because its pre-N segment could still function as a pseudosubstrate inhibitor.

Based on an AlphaFold3 model of a pre-N-CCDC134 complex, we made three point mutations (hereafter “RLS”) on one face of a predicted helical segment of the pre-N domain ~40 a.a. distal to the SRT pseudosubstrate site in HSP90B1 (fig.S7G). The RLS mutation increased HSP90B1 hyperglycosylation and also prevented suppression of HSP90B1 hyperglycosylation by CCDC134 (fig.S7H). However, in contrast to the T44A mutation, RLS resulted in the hyperglycosylation of only a fraction of HSP90B1, mimicking the phenotype observed when CCDC134 is lost (fig.S7H and Fig.2E). Accordingly, the phenotype of the T44A/RLS double mutation resembled that of the T44A single mutation (fig.S7H). These genetic studies support the view that suppression of HSP90B1 hyperglycosylation involves two ordered (but mutationally separable) steps: partial inhibition of N-glycosylation by the SRT pseudosubstrate site followed by recruitment of CCDC134, through interactions with both STT3A and the RLS region of the pre-N segment, to fully suppress N-glycosylation.

To investigate biochemical interactions between CCDC134, HSP90B1 and OST-A suggested by genetic experiments, we used a cell-free translation and translocation system that combines rabbit reticulocyte lysate (RRL) with microsomes isolated from HEK293T cells of any desired genotype (33). Reactions containing wild-type, *CCDC134*^-/-^, *STT3A*^-/-^ or *STT3B*^-/-^ microsomes were programmed with mRNA encoding the 1-93M model substrate derived from HSP90B1 (Fig.3F). As we observed in cells, N-glycosylation of 1-93M was enhanced when it was translated in the presence of microsomes lacking either CCDC134 or STT3A (Fig.3G) or when it contained the T44A mutation in the pseudosubstrate site (Fig.3H). Furthermore, we confirmed that N-glycosylation likely occurs co-translationally in *CCDC134*^-/-^ microsomes (fig.S8A and S8B) as it does in *CCDC134*^-/-^ cells (fig.S7B and S7C).

To assess protein interactions during translation and translocation, we used a FLAG-tagged 1-93M construct lacking a stop codon to stall translation, trapping the nascent chain in the ER translocon (Fig.3F). The stalled nascent chain was isolated using the FLAG tag and associated proteins detected by immunoblotting. As expected for a secretory ER translocon, STT3A, SEC61 and the ribosome were all pulled down with the stalled 1-93M nascent chain (Fig.3H) (34, 35). CCDC134 was also recruited to this translocon and its recruitment was dependent on (1) the presence of the 1-93 pre-N segment, (2) the integrity of the SRT pseudosubstrate site and (3) the presence of STT3A (Fig.3H, fig.S8C and S8D). Using stall substrates of various lengths (Fig.S8C), we found that CCDC134 was only recruited when the nascent chain was long enough so that the pre-N sequence of HSP90B1 would be exposed to the lumen of the ER (Fig.S8E). Resolving the stall using puromycin to release the nascent chain from its tRNA conjugate abolished the recruitment of CCDC134, showing that its recruitment requires an intact translocon and hence occurs co-translationally (fig.S8F). These experiment show that the recruitment of CCDC134 to the secretory translocon assembled by 1-93M in an *in vitro* reconstituted system is specific and has the same requirements (the pre-N segment, pseudosubstrate site and STT3A) as those needed to suppress HSP90B1 hyperglycosylation and promote cell-surface LRP6 trafficking and WNT signaling in intact cells. Separation-of-function mutagenesis (fig.S7D-S7H) shows that CCDC134 recruitment likely involves interactions with both STT3A and the pre-N segment since point mutations in either can prevent CCDC134 activity without disrupting other functions of these proteins.

### CCDC134 stabilizes a translocon-proximal protective scaffold for HSP90B1

The observation that an OST-A (whose main job is to N-glycosylate proteins) suppresses the N-glycosylation of a specific substrate (HSP90B1) is paradoxical. It suggests that OST-A may not function as an enzyme in this context, but rather as a scaffold.

**Figure 4:**
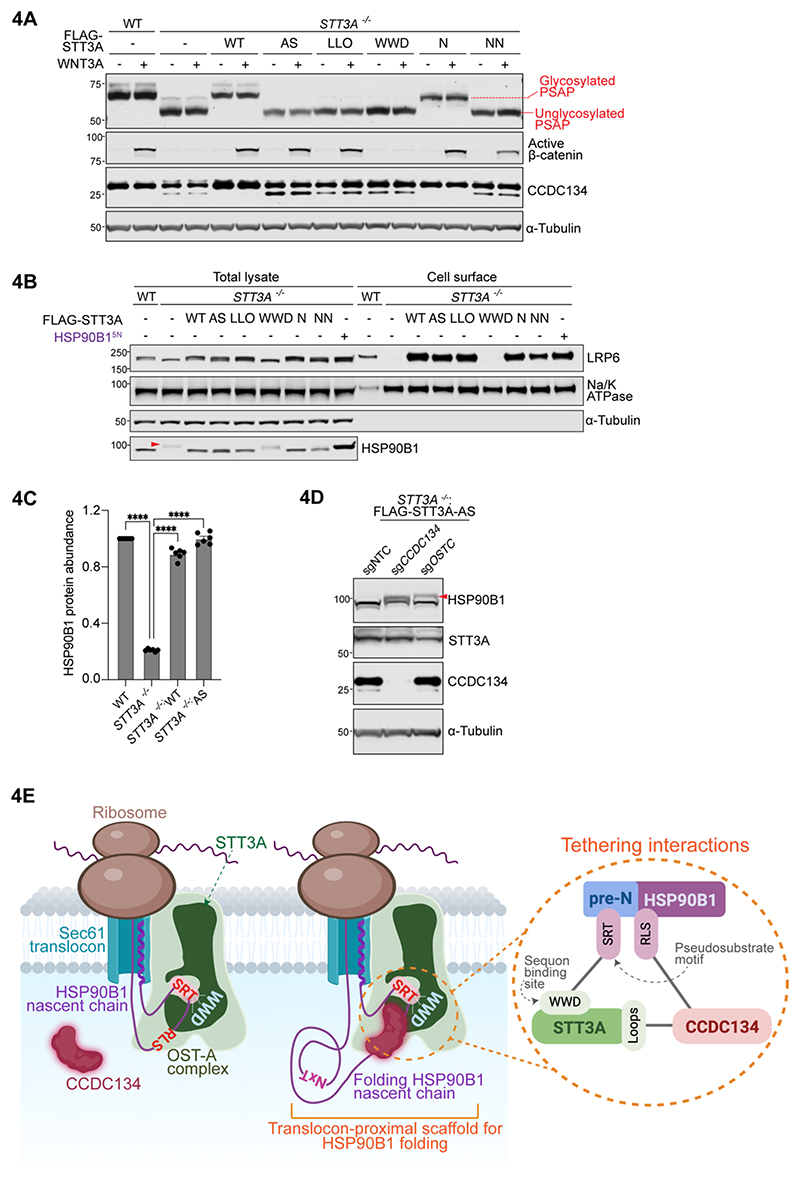
The oligosaccharyltransferase activity of OST-A is not required to promote HSP90B1 stability and WNT signaling. **(A**,**B)** Glycosylation status and abundances of PSAP (**A**), active β-catenin (**A**), CCDC134 (**A**), HSP90B1 **(B)** and cell-surface LRP6 (**B**) in wild-type (WT) cells or *STT3A*^*-/-*^ cells stably expressing FLAG-STT3A variants carrying mutations in various sites involved in catalytic transfer of the glycan from the lipid-linked oligosaccharide to the asparagine in sequons. Variants (shown on a structure in fig.S9A) carry mutations in residues involved in active site chemistry (AS), lipid-linked oligosaccharide binding (LLO), sequon binding (WWD) or N-glycosylation of STT3A itself (N and NN). Glycosylation of PSAP (**A**) was used to assess OST-A activity in cells. See fig.S9B. **(C)** HSP90B1 protein abundance in wild-type (WT) cells, *STT3A*^*-/-*^ cells, and *STT3A*^*-/-*^ cells stably expressing wild-type or catalytically inactive (AS) STT3A. The STT3A-AS variant carries mutations in four residues involved in the chemical step of glycan transfer (fig.S9A). Statistical significance was determined by one-way ANOVA with Dunnett’s multiple comparisons test; **** p<0.0001. **(D)** Abundance and glycosylation status (red arrowhead) of endogenous HSP90B1 in *STT3A*^*-/-*^ cells stably expressing (1) catalytically inactive FLAG-STT3A-AS carrying mutations in active site residues (fig.S9A) and (2) sgRNAs targeting CCDC134 or OSTC. sgNTC=non-targeting control sgRNA. **(E)** During translation, HSP90B1 is tethered to a specialized CCDC134-containing translocon that forms a specialized microenvironment for its folding. Tethering interactions are shown in the circular inset: CCDC134 interacts both with STT3A and HSP90B1, while the pre-N domain of HSP90B1 itself binds to the sequon binding site of STT3A. This translocon-proximal scaffold prevents STT3A from recognizing sequons in HSP90B1 and also sterically prevents access of these sequons to OST-B during folding. See fig.S10.

To test this possibility, we used *STT3A*^*-/-*^ cells to stably express STT3A variants carrying mutations in key residues known to be involved in binding to the lipid-linked oligosaccharide (LLO) (36, 37), binding to the N-X-S/T sequon (38), catalyzing the chemical step in LLO transfer to the carboxamide side chain of asparagine (active site residues) (39) and in N-glycosylation of STT3A itself (40) (fig.S9A). Unlike the complete loss of STT3A, these mutations do not compromise OST-A complex assembly or recruitment to the translocon. As expected, each set of mutations abolished the ability of STT3A to N-glycosylate PSAP (Fig.4A). Remarkably, inactive STT3A variants carrying mutations in the active site or LLO binding site were both fully able to suppress HSP90B1 hyperglycosylation (Fig.4B and fig.S9B), restore HSP90B1 abundance (Fig.4C), restore cell-surface levels of LRP6 (Fig.4B and fig.S9B), and rescue WNT signaling (Fig.4A). These inactive variants still depended on CCDC134 and the translocon adaptor subunit OSTC to inhibit HSP90B1 hyperglycosylation (Fig.4D). In contrast, the STT3A variant carrying mutations in the sequon binding site (STT3A-WWD, fig.S9A) was defective in suppressing HSP90B1 hyperglycosylation and restoring cell surface LRP6 (Fig.4A and 4B, fig.S9B). Thus, the capacity of STT3A to transfer glycans to proteins is not required for its ability to regulate HSP90B1 hyperglycosylation; however, its abilities to bind the SRT pseudosubstrate site and associate with the translocon are required.

We propose that the pre-N segment tethers HSP90B1 during its translation, translocation and folding to translocon-associated OST-A through an interaction between the SRT pseudosubstrate site and the STT3A sequon binding site (Fig.4E). Two consequences ensue: (1) OST-A is prevented from N-glycosylating subsequent sequons in HSP90B1 as they enter the ER lumen and (2) HSP90B1 is shielded from OST-B until it completes folding, rendering its facultative sequons inaccessible (Fig.4E and fig.S10). While the sequon-binding site is also conserved in STT3B, this model critically depends on the N- to C-terminal scanning mechanism of co-translational glycosylation, a feature unique to OST-A. We speculate that the association of OST-A with the translocon, through the dedicated subunits OSTC and KRTCAP2, enabled an expansion of its functions (beyond glycan transfer) to include serving as a protective folding microenvironment that can recruit ER factors to proteins being co-translationally transported across the ER membrane (35). More generally, our work supports the emerging view that custom translocons, tailored by information provided by the nascent chain, can serve as dynamic regulatory platforms to control the biogenesis and fate of secreted and membrane proteins (34, 41). In our case, the nascent chain recruits CCDC134 to an OST-A-containing translocon to regulate the N-glycosylation and stability of the ER chaperone HSP90B1, with direct consequences for WNT signaling sensitivity and tissue development.

CCDC134 functions as a substrate-specific inhibitor in our model, making contacts with both the substrate (pre-N segment) and the enzyme (STT3A) to prevent the N-glycosylation of HSP90B1 (Fig.4E **inset** and fig.S10). Evolutionary sequence analysis supports a shared function of these proteins. While HSP90B1 belongs to a clade that dates back to the base of the eukaryotic tree (fig.S11A), CCDC134 is a much later invention, found in animals and their sister groups (fig.S11B). It is only within the CCDC134-containing group of organisms that we observe significant constraints on the sequence of the HSP90B1 pre-N domain, including the pseudosubstrate site and surrounding sequence (Fig.S11B). Interestingly, fungi show a concomitant loss of both CCDC134 and sequence conservation in the pre-N segment (Fig.S11B).

An exciting prospect raised by our results is that translocon composition and function may be regulated by signals from the ER or the cytoplasm to regulate cell-cell communication in multicellular organisms. The client list of HSP90B1 is enriched in cell surface receptors and secreted ligands. Apart from LRP5/6, HSP90B1 facilitates the folding of Toll-like receptors, integrins, IGF1R and its ligand IGF1, the TGF-β docking receptor GARP and the platelet glycoprotein Ib complex (42–44). Indeed, a recent study found that *CCDC134* is required for the cell surface expression of TLR4, a receptor for bacterial endotoxins that activates inflammatory signaling through the NF-kB pathway (45). DepMap cluster analysis (fig.S7A) and prior work suggested a link between Insulin-like Growth Factor Receptor (IGF1R) and both HSP90B1 and STT3A (44, 46). Indeed, we found that cell surface expression of IGF1R (like LRP6) is disrupted by the loss of either CCDC134 or STT3A (fig.S12A-S12C). Expression of the HSP90B1^5N^ mutant bypassed this defect, implicating HSP90B1 hyperglycosylation. Thus, the CCDC134-OST-A-HSP90B1 system is not restricted to the regulation of WNT signaling but may play a broader role in other signaling systems that depend on HSP90B1 to facilitate folding of cell surface receptors. The co-essential relationships between HSP90B1, CCDC134 and OST-A across the DepMap panel of >1000 cancer cell lines (Fig.1A) are likely a consequence of their shared role in IGF1 signaling, known to be a universal driver of cell growth and survival (fig.S7A) (47).

Perhaps the most pressing question for the future is why such a system has evolved in animals to control signal reception at the cell surface and whether it can itself be regulated by other signals from inside or outside the cell. CCDC134 abundance is not regulated by either WNT or unfolded protein response pathways, suggesting that it is not a feedback regulator or directly responding to ER stress. One possibility is that CCDC134 and HSP90B1 serve to signal the biosynthetic capacity (or some other function) of the ER to the reception of cell surface signals that regulate proliferation or differentiation. Based on its link to the human disease OI (fig.S4), this regulatory mechanism may be particularly important in professional secretory cells like osteoblasts, where (WNT-dependent) differentiation must be coordinated with a massive expansion of the capacity of the ER to synthesize and N-glycosylate secreted components of the bone matrix.

## Supplementary Material

Supplementary Materials

## Data Availability

All unique reagents generated in this study are available from the Lead Contact, Rajat Rohatgi (rrohatgi@stanford.edu), and will be provided upon request. All data are available in the manuscript or the supplementary materials. This paper does not report original code. All FASTQ files from CRISPR/Cas9 screen have been deposited into the NIH Short Read Archive (SRA) with BioProject accession number PRJNA1087438. Raw data files for N-glycoproteomic analyses have been deposited into the MassIVE database under the accession number MSV000094280. All patient fibroblast cells used in this manuscript have been previously established by Dr. Valérie Cormier-Daire and reported in a prior peer-reviewed publication from her laboratory (PMID 32181939, J Bone Miner Res. 35(8) 1470-1480). As noted in this original publication, informed consent for participation and sample collection were obtained via protocols approved by the Necker-Enfants Malades Hospital, AP-HP, F-75015 Paris, France. This work used only these previously published unidentifiable or de-identified cell lines and did not involve any active patient recruitment or new sample collection from patients.

## One-Sentence Summary

N-glycosylation of asparagine residues on proteins can be regulated by specificity factors in the endoplasmic reticulum to control cell-surface signaling and tissue development.
