# Supplementary material for: Regulated N-glycosylation in the endoplasmic reticulum controls chaperone function and receptor trafficking
Source: Science. Author manuscript; Available in PMC 2025 Jan 24. (PMC7617332; doi:10.1126/science.adp7201)
Supplement: Supplementary Materials [file EMS202697-supplement-Supplementary_Materials.pdf]

Supplementary Materials for

Title: Regulated N-glycosylation in the endoplasmic reticulum controls chaperone function and receptor trafficking

Authors: Mengxiao Ma[1], Ramin Dubey[1], Annie Jen[4], Ganesh V. Pusapati[1], Bharti Singal[9], Evgenia Shishkova[2,3], Katherine A. Overmyer[3,4], Valérie Cormier-Daire[6], Juliette Fedry[8], L. Aravind[7], Joshua J. Coon[2,3,4,5], and Rajat Rohatgi*[1]

Affiliations:
[1]Departments of Biochemistry and Medicine, Stanford University School of Medicine, Stanford, CA 94305, USA.
[2]National Center for Quantitative Biology of Complex Systems, Madison, WI 53706, USA
[3]Morgridge Institute for Research, Madison, WI 53515, USA
[4]Department of Biomolecular Chemistry, University of Wisconsin, Madison, WI 53506, USA
[5]Department of Chemistry, University of Wisconsin, Madison, WI 53506, USA
[6]Université de Paris Cité, Génétique clinique, INSERM UMR 1163, Institut Imagine, Hôpital Necker-Enfants Malades (AP-HP), Paris, France
[7]National Center for Biotechnology Information, National Library of Medicine, National Institutes of Health, Bethesda, MD 20894, USA.
[8]MRC Laboratory of Molecular Biology, Cambridge, CB2 0QH, UK
[9]Stanford SLAC CryoEM Initiative, Stanford, CA 94305, USA

*Correspondence to rrohatgi@stanford.edu

The PDF file includes:
Materials and Methods
Figs. S1 to S12
References (*48-64*)

Other Supplementary Materials for this manuscript include the following:
Data S1. Results from genome-wide CRISPR/Cas9 knockout screens.
Data S2. Results from N-glycoproteomics.

MATERIALS AND METHODS

Constructs

CCDC134 constructs: *CCDC134* cDNA with a 3xHA tag inserted just after the ER signal sequence was ordered as a gene fragment from Twist Bioscience, cloned into the Gateway compatible entry vector pENTR2B Dual Selection Vector, and used as a template for generation of all *CCDC134* constructs. CCDC134 constructs were cloned into pEF5/FRT/V5-DEST (Thermo Fisher Scientific) or pLenti CMV PURO DEST (*50*) using Gateway recombination methods. Doxycycline inducible *CCDC134* constructs were cloned by PCR amplification followed by Gibson assembly (New England Biolabs) into pLenti-TRE-rtta3G-BLAST.

HSP90B1 constructs: pDONR223_HSP90B1_WT was purchased from Addgene (Plasmid #82130) and Gibson assembly was used to insert a 3xFLAG tag just after the ER signal sequence. All *HSP90B1* mutant and truncation constructs were generated using PCR amplification followed by Gibson assembly and cloned into pEF5/FRT/V5-DEST (Thermo Fisher Scientific, Invitrogen) or pLenti CMV PURO DEST (*50*) using Gateway methods. The following mutations were made in *HSP90B1*: HSP90B1^{1N} (T219I), HSP90B1^{5N} (S64A, S109A, S447A, T483I, T504I), HSP90B1 with ectopic buried (Bur3N) sequons (G164S, K493T, E659T), and HSP90B1 with ectopic exposed (Exp3N) sequons (L145T, M622T, Y678T).

STT3A constructs: STT3A-FLAG was a gift from Jan Carette (*51*) and used as a template to generate STT3A constructs and mutants.

PSAP constructs: *PSAP* cDNA was amplified from RKO cells using indicated primers (Fwd: 5'-ATGTACGCCCTCTTCCTCCTG-3', Rev: 5'-CTAGTTCCACACATGGCGTTTGC-3') and used as a template for generation all *PSAP* constructs.

All constructs were fully sequenced to confirm accuracy.

Reagents and antibodies

WNT3A conditioned media was produced as previously described (*11*). Briefly, 1.5 x 10^6 Wnt-3A cells or L cells were seeded in 15 cm tissue culture-treated dishes, grown for 3 days, media was replenished, and after an additional 3 days, conditioned media was collected, filtered through 0.2 µm polyethersulfone (PES) membrane filter, aliquoted, flash-frozen in liquid nitrogen, and stored at −80°C. Recombinant human IGF1 ligand was purchased from Gibco. Puromycin and blasticidin were purchased from Sigma-Aldrich. The transfection reagent polyethylenimine (PEI) was purchased from Polysciences and polybrene from MilliporeSigma. Bafilomycin A1 was purchased from Cayman Chemical, Bortezomib from LC labs, kifunensine from Tocris Bioscience, and NMS-873 from Sigma-Aldrich. Endoglycosidase H (EndoH) and Peptide-*N*-Glycosidase F (PNGaseF) were purchased from New England Biolabs. Phenylmethanesulfonyl fluoride (PMSF) and cycloheximide were purchased from Sigma-Aldrich, micrococcal nuclease from New England Biolabs, RNase-free DNase from Promega, SUPERaseIn from Invitrogen, and RNaseA from Gold Biotechnology. ANTI-FLAG® M2 Affinity Gel was purchased from Sigma-Aldrich. The following primary antibodies were used: mouse anti-CCDC134 (E-5, Santa Cruz Biotechnology, 1:500); mouse anti-HSP90B1 (H-10, Santa Cruz Biotechnology, 1:2000); mouse anti-LRP5 (B-9, Santa Cruz Biotechnology, 1:500); rabbit anti-LRP6 (C5C7, Cell Signaling Technology, 1:1000 for immunoblot); mouse anti-LRP6 ectodomain (clone A59, MilliporeSigma, 2ug/sample for cell surface staining); Alexa Fluor® 488 rabbit anti-Giantin (A488-114L, Covance, 1:500 for immunofluorescence); mouse anti-α-Tubulin (Clone DM1A, MilliporeSigma, 1:10000); rabbit anti-active (non-phosphorylated) β-catenin (D13A1, Cell Signaling Technology, 1:500); rabbit anti-Na/K ATPase (3010S, Cell Signaling Technology, 1:1000); rabbit anti-MESD (10958-1-AP, Proteintech, 1:1000); mouse anti-FLAG (clone M2, MilliporeSigma, 1:2000); rabbit anti-FLAG (F7425, Sigma-Aldrich, 1:2000); rabbit anti-PSAP (GTX101064, GeneTex, 1:1000); rabbit anti-STT3A (12034-1-AP, Proteintech, 1:1000); rabbit anti-STT3B (15323-1-AP, Proteintech, 1:1000); rabbit anti-OSTC (PA5-34060, Invitrogen, 1:1000);

mouse anti-RPL17 (C-8, Santa Cruz Biotechnology, 1:2000); rabbit anti-Sec61b (15087-1-AP, Proteintech, 1:1000); mouse anti-EMC3 (67205-1-Ig, Proteintech, 1:2000); rabbit anti-NOMO2 (14328-1-AP, Proteintech, 1:2000); rabbit anti-TRAPα (gift from Tino Pleiner, 1:2000); rabbit anti-IGF1R (D23H3, Cell Signaling Technology, 1:1000); rabbit anti-phospho-IGF1R (MA515148, Invitrogen, 1:1000); mouse anti-GAPDH (60004-1-Ig, Proteintech, 1:10,000). Secondary antibodies conjugated to horseradish peroxidase or Alexa Fluor dyes were obtained from Jackson Laboratories and Thermo Fisher Scientific. IRDye® 800CW Donkey anti-mouse and anti-rabbit IgG (H + L) were purchased from LI-COR.

Cell lines

L Wnt-3A (ATCC Cat. # CRL-2647), L cells (ATCC Cat. # CRL-2648), HEK293T (ATCC Cat. # CRL-3216), RKO cells (ATCC Cat. # CRL-2577), and C3H10T1/2 cells (ATCC Cat. # CCL-226) were grown in Dulbecco's Modified Eagle Medium (DMEM) containing high glucose (Cytiva) and supplemented with 10% fetal bovine serum (FBS) (Sigma-Aldrich), 1 mM sodium pyruvate (Gibco), 2 mM L-Glutamine (Gibco), 1x MEM non-essential amino acids solution (Gibco), penicillin (40 U/ml) and streptomycin (40 µg/ml) (Gibco), in a humidified atmosphere containing 5% CO_2 at 37°C. MC3T3-E1 cells (ATCC Cat. # CRL-2593) were grown in Alpha Minimum Essential Medium (aMEM) without ascorbic acid (Gibco Cat. # A1049001) supplemented with 10% FBS (Sigma-Aldrich), penicillin (40 U/ml) and streptomycin (40 µg/ml) (Gibco), in a humidified atmosphere containing 5% CO_2 at 37°C. Clonally derived *STT3A*$^{-/-}$ and *STT3B*$^{-/-}$ HAP1 cells were a gift from Jan Carette(*51*) and the wild-type HAP1 cell line from which pooled knockout lines were generated was a gift from Thijn Brummelkamp (now available from Horizon Discovery, Cambridge, United Kingdom). All HAP1 cells and derivatives thereof were grown in Iscove's Modified Dulbecco's Medium (IMDM) (Cytiva Cat. # SH30228FS) and supplemented with 10% FBS (Sigma-Aldrich), 2 mM L-Glutamine (Gibco), penicillin (40 U/ml) and streptomycin (40 µg/ml) (Gibco), in a humidified atmosphere containing 5% CO_2 at 37°C. Primary human fibroblasts were provided by Valerie Cormier-Daire and cultured as described previously(*28*).

Parent cell lines purchased from ATCC or Thermo Fisher Scientific (see above) came with a certificate of authentication from the vendor and were used without further validation. Patient fibroblasts from Dr. Cormier-Daire were validated by western blotting to ensure lack of CCDC134. HAP1 cells were validated by propidium iodide staining to ensure a haploid genome content. All stable or gene-edited cell lines derived from these parental cells were validated by western blotting or genomic PCR. Cell lines were confirmed to be negative for Mycoplasma infection when introduced into the lab (with the exception of patient fibroblasts).

Pooled genome-wide CRISPR/Cas9 screens

Generating a reporter cell line. A clonally derived RKO WNT EGFP reporter (RKO-7TG_scc-12) was generated as described previously (*11*). The clonal reporter line was transduced with Cas9 (lentiCas9-Blast; Addgene#52962) and a second round of clonal derivation was performed to generate multiple clonal cell lines that were analyzed for optimal Cas9 expression and WNT3A-induced EGFP reporter fluorescence. A clonal cell line (RKO-7TG_scc-12; Cas9_scc-7) with >90% on-target genome editing activity using two positive control sgRNAs (*CTNNB1* and *TCF7L2*) and also displayed the widest dynamic range for WNT3A-induced EGFP reporter fluorescence was selected as the reporter line for CRISPR/Cas9 knockout screens. Screen design was modeled after previous screens in the lab (*52*).
Generating knockout library and screen. The Brunello CRISPR library (Addgene #73178 (*53*)) was used to generate our genome-wide collection of mutant RKO cells. Brunello library amplification, lentiviral production, functional titer determination, and transduction were performed as described previously with minor modifications(*54*). Briefly, the Brunello library was amplified in Endura electrocompetent cells

(Lucigen) and subjected to Next-Generation Sequencing (NGS) to determine sgRNA distribution. For lentivirus production, 18 million 293FT cells were seeded in T225 flasks (40 flasks in total) and transfected the following day with 3.4 µg pMD2.G (Addgene #12259), 6.8 µg psPAX2 (Addgene#12260), and 13.6 µg lentiviral target (CRISPR) plasmid, and 195 µl of 1 mg/ml polyethylenimine (Polysciences) per flask. 48 hours after transfection, lentivirus was harvested, filtered through a 0.45 µm filter, aliquoted into multiple 50 ml tubes and stored at -80°C. The functional titer of the lentivirus was determined by surviving RKO cells after 24 hours of infection with virus followed by 48 hours of 2ug/mL puromycin treatment. RKO-Wnt reporter cell line stably expressing Cas9 was transduced with the Brunello library at a Multiplicity of Infection (MOI) =0.3 (360 million cells were transduced with virus to achieve ~1000 fold representation of each sgRNA) in the presence of 10 µg/ml polybrene. 24 hours after infection, cells were split and selected with puromycin (2 µg/ml) for seven days and frozen in aliquots of 5 million cells/vial. Genomic DNA (gDNA) was extracted from cells using Quick-gDNA Midiprep kit (Zymo Research) and subjected to NGS to determine sgRNA distribution. In all screens, 100 million cells were initially thawed into 5 x15 cm tissue culture-treated dishes and two days later split into a new set of 15 cm dishes such that at least 100 million cells were plated again to maintain 1000 fold representation of each sgRNA. On the fourth day the cells were split and 13 million cells were plated in 6 x 15 cm dishes. The next day the cells were treated with 50% WNT3A conditioned media for 24 hours. Cells were trypsinized and 4 million cells were pelleted and frozen (unsorted population) and the remaining ~40 million cells (corresponding to 500-fold representation of each sgRNA in the Brunello library) were sorted for cells with the lowest 10% of EGFP fluorescence. The screen was performed twice under identical conditions.

NGS sequencing and analysis. Genomic DNA (gDNA) was extracted from unsorted and sorted cells and the sgRNA library was amplified as described previously (*54*). Briefly, each sgRNA library PCR was set up with a mix of 10 NGS-Lib-Fwd primers ("staggered" Fwd primer mix to increase library diversity) and a unique NGS-Lib-Rev primer with the entire gDNA (5 µg per 100 µL reaction). The PCR product was purified, quantified by qRT-PCR and subjected to sequencing on Illumina HiSeq. In all the screens, we averaged >100 reads per sgRNA in the library. For analysis, reads from the FASTQ files generated by sequencing were tallied for each guide by taking the first 20 base-pairs from each read (that were trimmed to remove adapter and vector backbone sequences) and mapping that sequence to the identical sgRNA sequence. For each screen, a table of reads per guide that includes the counts from the sorted and unsorted populations from both replicates was generated. The tables generated from the two independent duplicates of each screen were analyzed together by the MAGeCK computational tool (*55*), specifying the 1000 control sgRNAs for normalization and generation of the null distribution for MAGeCK with the "--control-sgrna" option and computing the log fold change for the gene using the mean of all of the guides for a given gene with the "--gene-lfc-method mean" option.

Immunoblot analysis

For immunoblot analysis of β-catenin in RKO cells, whole cell lysates were prepared in β-catenin lysis buffer (*56*): 30 mM Tris at pH 7.4, 150 mM NaCl, 1% Triton X-100, 0.5 mM TCEP, 1 mM EDTA, 10% glycerol, 1mM NaF, 1 mM $Na_3VO_4$1, and 1x SIGMAFAST protease inhibitor cocktail (MilliporeSigma). For all other immunoblotting data presented in the manuscript, whole cell lysates were prepared in RIPA lysis buffer: 50 mM Tris at pH 8.0, 150 mM NaCl, 2% NP-40, 0.25% Deoxycholate, 0.1% SDS, 0.5 mM TCEP, 10% glycerol, 1x SIGMAFAST protease inhibitor cocktail (MilliporeSigma), and 1x PhosSTOP phosphatase inhibitor cocktail (Roche). For resolving LRP6 and full length HSP90B1 glycoforms, samples were run on a 7% Tris-glycine gel. For resolving 1-93M glycoforms, samples were run on a 9% Tris-glycine gel. The resolved proteins were transferred onto nitrocellulose membrane (Bio-Rad Laboratories) using a wet electroblotting system (Bio-Rad Laboratories) followed by immunoblotting.

Flow cytometry analysis

For analysis of the WNT EGFP reporter, cells were treated with either WNT3A conditioned media or control conditioned media for 24 hours, trypsinized, filtered through 70 µm sterile cell strainer (Falcon), and EGFP fluorescence was measured immediately on a Sony SH800 flow cytometer. Fluorescence data for 10,000 singlet-gated cells was collected and subsampled thrice, each data point represents median reporter fluorescence from each subsampled population.

For cell surface staining of LRP6 in primary fibroblasts, cells were harvested by brief incubation with trypsin (3-4 min), immediately resuspended in complete media and counted. The cells were pelleted and resuspended in Staining Buffer or SB (10% FBS and 0.05% sodium azide prepared in PBS) at a concentration of 0.5 million cells/100 µL SB. 100 µL 10% donkey serum was added to 100 µL cell suspension and the cells were blocked for 10 min at room temperature. Cells were pelleted, resuspended in 100 µL fresh SB and stained with 2 µg of an anti-LRP6 antibody (A59, MilliporeSigma) for 30 min on ice. Cells were washed twice, resuspended in 100 µL fresh SB and then incubated for 30 min on ice in 1 ug of donkey anti-mouse IgG, Alexa Fluor 647 (Thermo Fisher Scientific). Cells were finally washed twice, resuspended in 100 µL fresh SB and analyzed on a Sony SH800 flow cytometer. Fluorescence data for 2,000 singlet-gated cells was analyzed per experiment and the experiment was repeated thrice. For all other experiments, fluorescence data for 10,000 singlet-gated cells was collected unless indicated otherwise.

Immunofluorescence analysis

RKO cells expressing doxycycline-inducible 3xHA tagged CCDC134 variants were seeded on coverslips, grown for 1 day, and treated with 25 nM doxycycline for 24 hours. Coverslips were washed with PBS, fixed with 4% (w/v) paraformaldehyde (PFA) at room temperature, permeabilized and blocked with PBS containing 1% donkey serum + 1% BSA + 0.1% Triton X-100 for 1 hour at 4°C, and incubated with primary antibodies (mouse anti-CCDC134 at 1:200 and rabbit anti-Giantin-AF488 at 1:500) for 1 hour at 4°C. Coverslips were washed and incubated with secondary antibody (donkey anti-mouse AF647), mounted in ProLong Diamond with DAPI (Invitrogen P36962), and images were collected using an Olympus IX83 epifluorescence microscope equipped with an Orca Fusion scMOS camera using a x100 oil objective (NA 1.45).

Generation of clonally derived knockout cell lines

Clonal knockout RKO and HEK293T cell lines were generated using a dual sgRNA strategy as previously described (*52*). Briefly, two sgRNAs targeting candidate genes (or non-targeting control, NTC) 200-800 bases apart were designed using the Synthego Knockout Guide Design tool (http://design.synthego.com) and cloned into pSpCas9(BB)-2A-GFP (PX458; Addgene #48138) and pSpCas9(BB)-2A-mCherry, the latter generated by replacing the GFP cassette in PX458 with mCherry. Four days after co-nucleofection in RKO cells (Nucleofector 2b device using program A-024 and Lonza Cell Line Nucleofector® Kit V #VCA-1003) or co-transfection in HEK293T cells (X-tremeGENE9, Roche), GFP and mCherry double positive single cells were sorted into a 96-well plate using a Sony SH800 flow cytometer. Clonal lines were first screened by PCR to detect excision of the genomic DNA between the two sgRNA cut sites and further confirmed by immunoblot analysis using commercially available antibodies.

Generation of CRISPR/Cas9-mediated pooled knockout cell lines

For validation of candidate genes from CRISPR/Cas9 screen, two independent guides were designed and individually cloned into lentiCRISPR v2 plasmid (Addgene #52961) (*57*). Lentivirus was produced as

described above and used to infect RKO and HEK293T WNT-GFP reporter cells, followed by selection with puromycin (2 µg/mL) for 5 days. Pooled cell lines were analyzed by FACS for EGFP fluorescence after treatment with WNT3A or control conditioned media. For generation of pooled knockouts targeting all other genes in HAP1, MC3T3, and C3H10T1/2 cells, sgRNAs were selected from either the Brunello (human) or Brie (mouse) libraries and cloned into either lentiCRISPR v2 or lentiCRISPR v2-Blast (Addgene #83480). Lentivirus was produced and used to infect indicated cell lines, followed by selection with puromycin (2 µg/mL) or blasticidin (10 µg/mL).

Generation of stable cell lines expressing transgenes

Stable addback cell lines expressing tagged CCDC134, tagged HSP90B1, or tagged STT3A were generated using the lentiviral expression system. To generate virus, 700,000 HEK293T cells were seeded onto a 6-well plate and 24 hours later transfected with 200 ng pMD2.G (Addgene), 400 ng psPAX2 (Addgene), and 800 ng of the desired pLenti CMV Puro DEST or pLenti-TRE-rtta3G-BLAST construct using 7 µl of 1mg/ml polyethylenimine (PEI) (Polysciences). Approximately 48 hours post transfection, lentivirus conditioned media was harvested and filtered through a 0.45 µm filter. 0.5 ml of the filtered lentivirus solution was mixed with 1.5 ml of complete media containing 8 µg/mL polybrene (MilliporeSigma). The diluted virus was then added to the indicated cells seeded on 6-well plates. Approximately 24 hours post infection, cells were split and selected with puromycin (2 µg/mL) or blasticidin (10 µg/mL) for 3-7 days or until all the cells on the control plate are dead. For doxycycline inducible expression of 3xHA-CCDC134, cells were grown for 24 hours in a range of doxycycline concentrations with 5 nM inducing low, near-endogenous expression levels.

Cell surface biotinylation assay

RKO cell lines were seeded at a density of 2 x 10^6 on 10 cm tissue culture-treated dishes and grown for 2 days. Cell culture plates were removed from the 37°C incubator and placed on an ice-chilled metal rack in a 4°C cold room. Growth medium was removed and cells were quickly washed thrice with ice-cold DPBS+ buffer (1.47 mM KH_2PO_4, 8.06 mM Na_2HPO_4, 137.93 mM NaCl, 2.67 mM KCl, 0.9 mM $CaCl_2$, 0.49 mM $MgCl_2.6H_2O$, 5.56 mM dextrose, and 0.33 mM sodium pyruvate). Cells were incubated with a freshly prepared solution of 0.4 mM Sulfo-NHS-SS-Biotin (Thermo Fisher Scientific) in DPBS+ buffer for 30 min. Unreacted Sulfo-NHS-SS-Biotin was quenched with Tris pH 7.4 at 50 mM for 10 min. Cells were then washed thrice with 1x Tris-buffered saline (25 mM Tris-HCl pH 7.4, 137 mM NaCl, and 2.7 mM KCl) and whole cell extracts were prepared in a buffer containing 50 mM Tris-HCl pH-7.4, 150 mM NaCl, 2% NP-40, 0.25% deoxycholate, 1x SIGMAFAST protease inhibitor cocktail (MilliporeSigma), and 1x PhosSTOP phosphatase inhibitor cocktail (Roche). Biotinylated proteins from clarified supernatants were captured on a streptavidin agarose resin (Solulink), washed, eluted in NuPAGE-LDS sample buffer containing 100 mM DTT at 42°C for 30 min to cleave and release biotinylated proteins, and assayed by immunoblotting.

LC-MS/MS Proteomics

Peptide Preparation. Cell pellets were removed from -80 °C, where they were maintained prior to analysis. 1 mL of 5.4 M guanidine hydrochloride in 100 mM Tris HCl, pH 8.0, was added to each cell pellet. Samples were gently vortexed, then probe sonicated for 10 seconds to ensure cell pellet resuspension. A bicinchoninic acid (BCA) protein assay (Pierce, Rockford, IL) was performed according to manufacturer's instructions to determine the protein concentrations. 1 mg of protein from each cell pellet was transferred into separate 1.5 mL microcentrifuge polypropylene tubes. Sufficient LC-MS grade methanol was added to each sample to bring each sample to 90% volume/volume methanol in composition, then vortexed for 10 seconds. Each sample was then centrifuged at 9,000 x g for 5 minutes

at 5 °C to pellet the protein. After the supernatants were carefully decanted to waste. Each pellet was resuspended in freshly prepared 8M urea, 100 mM Tris HCl pH 8.0, 10 mM TCEP, 40 mM 2-chloroacetamide and vortexed for not less than 15 minutes at ambient temperature to resolubilize the protein. 20 µL of 1 mg/mL LysC prepared per manufacturer's instruction (VWR, Radnor, PA) was added to each 1 mg protein sample, then allowed to incubate at ambient temperature for four hours while gently rocking. Samples were then diluted with freshly prepared 100 mM Tris HCl pH 8.0 to reach a final urea concentration of 2 M, after which 20 µL 1 mg/mL trypsin (Promega, Madison, WI) was added to each sample. Samples were incubated at ambient temperature overnight while gently rocking. To stop digestion, 20 µL TFA was added to each sample, after which samples were centrifuged at 9,000 x g for 5 minutes to pellet insoluble material. The resulting supernatant was desalted using Strata-X 33 µm polymeric reversed phase SPE cartridges (Phenomonex, Torrance, CA).

Glycopeptide Enrichment. The desalted peptides were dried down in a vacuum centrifuge (Thermo Fisher Scientific, Waltham, MA). For glycopeptide enrichment, 1 mg desalted peptides were resuspended in 90% acetonitrile in 1% TFA, then placed onto 10 mg SOLA SPE columns (Thermo Scientific). The peptides were enriched for N-glycopeptides according to the published protocol (*58*) without the use of the vacuum manifold. The resulting enriched mixture was dried down in a vacuum centrifuge and resuspended in 20 µL 0.2% formic acid in water for analysis.

Instrument Analysis. Sample analysis was performed using a Vanquish Neo HPLC system coupled to an Orbitrap Ascend Tribrid mass spectrometer (Thermo Scientific, San Jose, CA). Mobile phase A was water with 0.2% formic acid, and mobile phase B was 80:20 v/v ACN:H2O with 0.2% formic acid. The gradient elution was carried out with a flowrate at 0.300 µL/min. 1 µg of enriched peptides (N-glycoproteomics) or unenriched tryptic peptides (shotgun proteomics) were loaded onto a 75 µm i.d. column with 1.7 µm, 130 Å pore size, Bridged Ethylene Hybrid (BEH) C18 particles (Waters, Milford, MA), packed in-house to a length of 30 cm (*59*). The column was heated to 50 °C during analysis.

For N-glycoproteomics mass spectrometer analysis, positive mode ionization was used. MS1 scans were acquired from 0 to 90 minutes every second at a scan range of 300-2,000 m/z, with a resolution of 60,000 in the Orbitrap and maximum injection time of 123 ms; normalized AGC target (%) was set to 250, equivalent to 1e6 ions, and RF lens (%) set to 30. Precursor ions with charge states 2-6 were isolated from a 1.3 Da window in the quadrupole with a dynamic exclusion period of 20 seconds. Data-dependence HCD MS2 scans with 36% normalized collision energy, maximum injection time of 59 ms, and a normalized AGC target of 200% (equivalent to 1e5 ions) were acquired in the Orbitrap at a resolution of 30,000 for precursors with the defined first mass of 150 m/z, and scanned for trigger ions of 204.0867, 138.0545, 366.1396, 274.0921, 292.1027, 126.055, 144.0655, 168.0654 or 186.076 (± 10 ppm). If trigger ions were detected, the precursor was re-isolated and fragmented with sceHCD of 35±15 over the scan range of 150-4,000 with the resolution of 30k and max injection time of 100 ms(*60*).

For shotgun proteomics mass spectrometer analysis, with positive mode ionization, MS1 scans were collected every second in the Orbitrap with a scan range of 300-1,350 m/z from 0 to 90 minutes, with a resolution of 240,000, a maximum injection time of 50 ms, RF lens (%) of 30, and a normalized AGC target (%) of 250, equivalent to 1e6 ions. Precursor ions with charge states 2- 5 were isolated in the quadrupole with an isolation window of 0.5 m/z. HCD MS2 scans were acquired in a data-dependent manner using a fixed normalized collision energy of 25% and an AGC target (%) of 250, equivalent to 2.5e4 ions, and collected in the ion trap from 150-1,350 m/z, with a maximum injection time of 14 ms and a dynamic exclusion period set to 10 seconds.

Data Analysis. For shotgun proteomics data, raw proteomic data files were processed by MaxQuant version 2.4.7.0 (*61*). The UniProt database of reviewed proteins and isoforms from Homo sapiens was retrieved on December 13, 2023. Default MaxQuant parameters were used for processing, along with the

following parameters: label-free quantification (LFQ) calculated with a minimum ratio of 1; match between runs enabled; MS/MS not required for LFQ comparisons. In the generated MaxQuant data output, protein identifications were removed that were indicated to be identified by site only, corresponded to reverse sequences, and/or to be potential contaminants by MaxQuant. Protein identifications that generated an intensity value of zero in 50% or more of the analyzed samples were also removed. Missing quantitative values among the remaining protein groups were imputed, log2-transformed, and statistically analyzed using Argonaut (*62*).

For N-glycoproteomics data, raw proteomic data files were processed by MSFragger-Glyco (v20.0) (*63*), using the UniProt database of reviewed proteins and isoforms from Homo sapiens retrieved August 31, 2023. Default search parameters for “Glyco-N-LFQ” workflow were used, except the range for peptides was expanded to 65 amino acids and max mass of 6,500; MaxLFQ quantification and match-between-runs were enabled. The number of missing values (zeroes) were counted across the samples in each condition; glycan-bearing peptide IDs were kept if there were intensity values above zero in at least three values within a single condition. Missing quantitative values among the remaining protein groups were imputed, log2-transformed, and statistically analyzed using Argonaut5.

Quantitative RT-PCR analysis

Approximately 48 hours before treatment, fibroblasts were seeded in 24-well plates at a density of 1.5 × 10^4 per well. Cells were treated for 12 hours with WNT3A or control conditioned media as indicated. Cells were harvested in 800 µl of TRIzol Reagent (Thermo Fisher Scientific Cat. # 15596018) and RNA prepared following manufacturer protocol. 250 ng of RNA was used to synthesize cDNA using iScript Reverse Transcription Supermix (Bio-Rad Laboratories Cat. # 170–8841) following manufacturer protocol. cDNA was diluted 1:100 in water, and 5 µl were mixed with 5 µl of *Power* SYBR™ Green PCR Master Mix (Applied Biosystems Cat. # 4367659) containing 200 nM each of forward and reverse primer for *AXIN2* (Fwd: 5’-GTCCAGCAAAACTCTGAGGG-3’, Rev: 5’-CTGGTGCAAAGACATAGCCA-3’), *TNFRSF19* (Fwd: 5’- GGTGCATTCTGCAGCCAGTCTT-3’, Rev: 5’- CAGGCATCTGAAAACTCGCCAC-3’), *GAPDH* (Fwd: 5’- AAAGGGTCATCATCTCTG-3’, Rev: 5’- GCTGTTGTCATACTTCTC-3’). Triplicate reactions for each cDNA and primer pair were run on a QuantStudio 5 Real-Time PCR System (Thermo Fisher Scientific) and transcript levels relative to *GAPDH* were calculated using the ΔCt method.

Preparation of rough microsomes

The original method (*64*) for isolation of microsomal membranes for cotranslational protein translocation from canine pancreas has been adapted to HEK293T cells (*33*). HEK293T cells were grown to ~80% confluency in 15 cm tissue culture-treated dishes, washed thrice with ~20 mL ice-cold PBS and collected by scraping in 5-10 mL of PBS. Cells were centrifuged for 5 min at 500 x g and resuspended in 2 mL lysis buffer (10 mM HEPES-NaOH pH 7.4, 250 mM sucrose, 2 mM $MgCl_2$, 0.5 mM DTT, 1x SIGMAFAST protease inhibitor cocktail) per dish of cells. Cells were homogenized using a chilled and equilibrated Isobiotec Cell Homogenizer (5–10 single passes, 14 µm clearance) on ice and lysate was cleared twice (1,500 x g for 3 min at 4°C). Microsomes were pelleted (10,000 x g for 10 min at 4°C) and resuspended in microsome buffer (10 mM HEPES-NaOH pH 7.4, 250 mM sucrose, 1 mM $MgCl_2$, 0.5 mM DTT) at a density of 1 mL buffer for every 3-4 dishes of cells. Each 1 mL aliquot of microsomes was treated for 10 min at 37°C with 4000 U micrococcal nuclease (New England Biolabs), 2 U RNase-free DNase (Promega), 1 mM $CaCl_2$, and 0.5 mM PMSF (Sigma-Aldrich), followed by quenching with 2 mM EGTA. Microsomes were pelleted (10,000 x g for 10 min at 4°C), resuspended in 1 mL microsome buffer containing 40 U SUPERaseIn and 0.1 mM EGTA, and pelleted again (10,000 x g for 10 min at 4°C). The membrane pellet was resuspended in fresh microsome buffer and adjusted to an absorbance at 260 nm of 50-75 and used fresh in translation reactions.

In vitro translation (IVT) and glycosylation analysis

RNA encoding indicated HSP90B1 nascent chains were prepared from PCR amplified and purified DNA template containing a T7 promoter and carried out at 37°C for 2 hours using the HiScribe® T7 High Yield RNA Synthesis Kit (New England Biolabs) and purified using the RNeasy Mini Kit (Qiagen). For *in vitro* glycosylation analysis of full length protein products, 50 µL IVT reactions were carried out for 2 hours at 30°C using commercially available Nuclease-Treated Rabbit Reticulocyte Lysate (RRL, Promega) and reaction components were added in the following order: 35 µL RRL, 1 µL of 1 mM complete amino acid mixture (combine amino acid mixtures provided with RRL), 5 µL microsomal membranes, 2 µg RNA, and nuclease-free water. IVT reaction was added immediately to 200 µL of immunoprecipitation buffer (50 mM Tris pH 7.4, 150 mM NaCl, 1% NP-40, 10% glycerol) containing 10 µL anti-FLAG M2 resin. For *in vitro* glycosylation analysis of translation intermediates, 200 µL IVT reactions were set up as described above and 50 µL was removed at each time point and added to 200 µL immunoprecipitation buffer containing 0.5 mM puromycin and 10 µL anti-FLAG M2 resin. Reactions were incubated with anti-FLAG M2 resin for 2 hours at 4°C, washed 3 times with immunoprecipitation buffer, and eluted twice in immunoprecipitation buffer containing 1 mg/mL 3xFLAG peptide (Sigma-Aldrich) (25 µL at 4°C overnight and 25 µL for 30 min at room temperature). Eluates were combined, applied to equilibrated Ultrafree-MC Centrifugal Filter (MilliporeSigma), and spun at 12,000 x g for 4 min at 4°C. Half of eluate was treated with Endo H prior to immunoblot analysis.

In vitro translation (IVT) and immunoprecipitation of ribosome nascent chain complexes

Templates for synthesis of stalled HSP90B1 nascent chains were PCR amplified using reverse primers encoding a terminal Met-Leu-Lys-Val (5′-CACCTTGAGCAT-3’) sequence and lacking a stop codon. RNA was prepared as described above. Translation reactions of 500 µL containing 60 µL microsomal membranes was prepared as described above and carried out for 1 hour at 30°C. Reactions were immediately diluted in cold 500 µL IVT stop buffer (50 mM HEPES pH 7.4, 200 mM NaCl, 10 mM $MgCl_2$) and centrifuged at 12,500 x g for 10 min, 4°C. Membrane pellet was washed once with 1 mL IVT stop buffer and centrifuged at 12,500 x g for 10 min, 4°C. Membrane pellet was resuspended in 500 µL IVT stop buffer and treated for 20 min at room temperature with 5000 U micrococcal nuclease, 1 mM $CaCl_2$, and 0.6 mM PMSF, followed by quenching with 2.5 mM EGTA, and pelleted at 12,500 x g for 10 min, 4°C. Membrane pellet was solubilized in 200 µL solubilization buffer (50 mM HEPES-KOH pH 7.4, 250 mM sucrose, 250 mM KOAc, 10 mM $MgCl_2$, 2.5% digitonin) for 45 min, rotating at 4°C, followed by addition of 200 µL of dilution buffer (50 mM HEPES-KOH pH 7.4, 250 mM sucrose, 150 mM KOAc, 10 mM $MgCl_2$), and cleared at 12,500 x g for 15 min, 4°C. Cleared supernatant was incubated overnight with 15 µL anti-FLAG M2 resin. Resin was washed thrice with 400 µL wash buffer (50 mM HEPES-KOH pH 7.4, 250 mM sucrose, 200 mM KOAc, 10 mM $MgCl_2$, 0.4% digitonin) and eluted twice in IVT stop buffer containing 1 mg/mL 3xFLAG peptide and 0.4% digitonin by rotating for 30 min at 4°C. For puromycin release of nascent chain, resin was incubated with 0.5 mM puromycin before elution and washed twice after puromycin treatment before proceeding with elution. Eluates were combined, applied to equilibrated Ultrafree-MC Centrifugal Filter (MilliporeSigma), and spun at 12,000 x g for 4 min at 4°C. For RNaseA and Endo H treatments, 15 µL of eluate was first treated with 50 µg/mL RNaseA, 10 mM EDTA, 0.5% SDS in a total of 20 µL for 15 min at 37°C. Then 1 µL of 1 M DTT was added and the sample boiled for 10 min, then 1 µL Endo H and 2.5 µL GlycoBuffer 3 (New England Biolabs) was added and incubated for 1 hour at 37°C, followed by immunoblot analysis.

Pulse chase analysis

Clonally derived *HSP90B1*$^{-/-}$ and *HSP90B1*$^{-/-}$; *CCDC134*$^{-/-}$ RKO cells stably expressing 3xFLAG-HSP90B1 were seeded at a density of 2 × 10^6 per 6 cm tissue culture-treated dish and grown for two days. Cells were washed twice with PBS and changed into starvation medium: Met/Cys-free DMEM (Gibco Cat. # 21013024) containing 10% dialyzed FBS for 40 min. For pulse labeling, cells were changed into 1 mL of starvation media containing 200 µCi/mL EasyTag EXPRESS^{35}S Protein Labeling Mix (PerkinElmer NEG772002MC), grown for indicated time points, immediately changed into ice-cold PBS and collected on ice. For pulse chase analysis, cells were pulse labeled as described and immediately changed into chase medium (complete DMEM containing an additional 2 mM Met and 2 mM Cys and 100 µg/mL cycloheximide) for the indicated time points and collected in ice-cold PBS. Whole cell lysate was prepared in lysis buffer (50 mM Tris at pH 7.4, 150 mM NaCl, 1% NP-40, 0.25% Deoxycholate, 10% glycerol, 1x SIGMAFAST protease inhibitor cocktail (MilliporeSigma), and 1x PhosSTOP phosphatase inhibitor cocktail (Roche)) and a small aliquot was used in LSC analysis to ensure equivalent CPMs of lysates were used for immunoprecipitation (generally 300-800 µg). Lysates were incubated with 10 µL anti-FLAG M2 resin for 2 hours at 4°C and washed 3 times with lysis buffer. 25% of the sample was used for Endo H (New England Biolabs) treatment following manufacturer protocol. All samples were eluted in NuPAGE-LDS sample buffer containing 100 mM DTT or 50 mM TCEP for SDS-PAGE analysis and transferred onto PVDF membrane (Bio-Rad Laboratories Cat. #162-0177). Dried membranes were exposed to a phosphor screen (Molecular Dynamics), scanned on Typhoon FLA 9500, and quantified in ImageStudio (LI-COR) to determine the distribution of glycoforms.

IGF signaling assay

HEK293T cell lines were seeded at a density of 1 × 10^6 cells per well in a 6-well tissue culture-treated dish and grown for 1 day. Cells were serum starved for ~20 hours and subsequently treated with human IGF1 ligand (PeproTech) in starvation media for 30 minutes. Cells were collected immediately on ice using ice-cold PBS and analyzed by immunoblot.

Evolutionary sequence analysis

Human CCDC134 9 (NP_079097.1) was extracted from the National Center for Biotechnology Information (NCBI) Genbank database. Sequence similarity searches were performed using it as the query with the PSI-BLAST program against the NCBI non-redundant (nr) database or the same database clustered down to 50% sequence identity using the MMseqs program with a profile-inclusion threshold was set at an e-value of 0.01. Profile-profile searches were performed with the HHpred program. Multiple sequence alignments (MSAs) were constructed using the MAFFT program. Sequence logos were constructed using these alignments with the ggseqlogo library for the R language. Signal peptides were predicted using a deep neural network as implemented in SignalP 6.

Evolutionary structure analysis

The JPred program was used to predict secondary structures using MSAs (see above). Sequence complexity analysis was performed using the SEG program. Structural models were generated using the RoseTTAfold and Alphafold2 programs. Multiple alignments of related sequences (>30% similarity) were used to initiate HHpred searches to be used by the neural networks deployed by these programs. Structures were rendered, compared, and superimposed using the Mol* program.

Comparative genomics and phylogenetic analysis

Clustering of protein sequences was performed using MMSEQS with empirical adjustment of the length of aligned regions and bit-score density threshold. Phylogenetic analysis was performed using the

maximum-likelihood method with the WAG substitution matrix and 20 gamma-distributed categories with the IQTree program.

Statistical analysis

Data analysis and visualization were performed in GraphPad Prism 10. Model figures were made with Biorender.com and all other figures were made in Adobe Illustrator 2022. The predicted human CCDC134 structure and predicted interaction between CCDC134 and HSP90B1 pre-N segment were generated using AlphaFold.

The one-way ANOVA test with Sidak's multiple comparison or Dunnett's multiple comparison were used to compare three or more groups with one independent variable. A two-way ANOVA test with Dunnett's multiple comparison or Tukey's multiple comparison were used to compare three or more groups with two independent variables. All comparisons shown were prespecified. All experiments were performed at least three different times with similar results. We note that a small sample size (*n*=3) makes it difficult to assess whether the variance between different samples is comparable. Throughout the paper, the *p*-values for the comparisons from GraphPad Prism 10 are denoted on on the graphs according to the following key: **** p-value<0.0001, *** p-value<0.001, ** p-value<0.01, * p-value<0.05, and n.s. Non-significant. Descriptions of replicates are included in figure legends.

Genome-wide loss-of-function CRISPR/Cas9 screens were performed twice under independent conditions and the duplicates from each screen were analyzed together using the MAGeCK tool (**fig.S1B**). Mass spectrometry analysis used six individual replicates (each replicate is an individual cell pellet processed in a different mass spectrometry run) and error bars depict standard error of the mean where indicated.

fig. S1

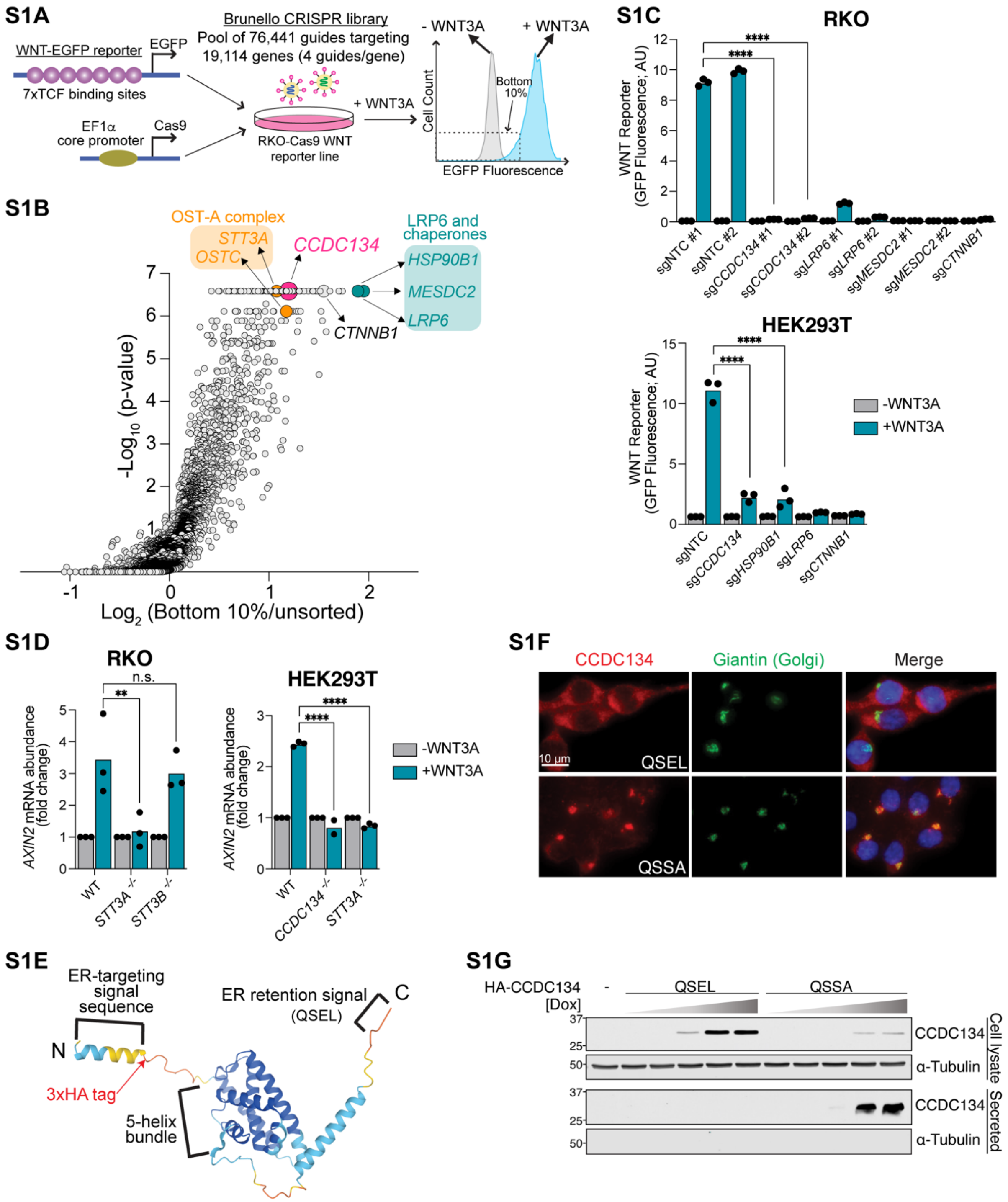

Supplementary Figure 1. CCDC134 is an ER protein that functions as a positive regulator of WNT signaling.

(A) Overview of a genome-wide loss-of-function CRISPR/Cas9 screen to identify positive regulators of WNT signaling induced by the ligand WNT3A (*11*). The screen was conducted in a human colon cancer cell line (RKO) engineered to express a WNT3A responsive fluorescent transcriptional reporter (WNT-EGFP) and Cas9. The mutant library was exposed to WNT3A and cells expressing sgRNAs that

impaired reporter activation were isolated by FACS using a gate that encompassed the bottom 10% of the reporter fluorescence distribution after WNT3A treatment (right).

(B) Volcano plot of CRISPR/Cas9 screen results. Each circle is one gene, the x-axis shows its enrichment or depletion calculated as the mean of all four sgRNAs targeting the gene in the bottom 10% sorted population relative to the unsorted population, and the y-axis shows statistical significance as measured by *p*-value. Genes that are the focus of this study are highlighted in color. *CTNNB1*, which encodes β-catenin, is highlighted as a positive control. LRP6 is a co-receptor for WNT ligands. HSP90B1 and MESD (encoded by the gene *MESCD2*) are ER chaperones that facilitate LRP6 folding. Two components of the OST-A complex (OSTC and STT3A) and CCDC134 were identified as novel positive regulators not previously linked to WNT signaling. Full screen results provided in **Data S1**.

(C) WNT-EGFP reporter fluorescence (+/- WNT3A) in RKO (top) or HEK293T cells (bottom) expressing a non-targeting control sgRNA (NTC) or sgRNAs (different from those used for the CRISPR/Cas9 screen in **fig.S1B**) targeting selected screen hits from (**B**). Each data point represents median WNT-EGFP reporter fluorescence from one population and bars show the mean of three populations. Statistical significance was determined by one-way ANOVA Sidak's multiple comparisons test; **** $p<0.0001$ (n=3, ~3300 cells each).

(D) Endogenous *AXIN2* mRNA abundance, measured by quantitative Reverse Transcription PCR (qRT-PCR), was used as a metric to assess WNT signaling strength in cells of the indicated genotypes. WNT signaling directly activates *AXIN2* transcription. WT: wild-type. Statistical significance was determined by two-way ANOVA with Dunnett's multiple comparisons test; ** $p<0.01$, **** $p<0.0001$.

(E) AlphaFold prediction of the structure adopted by CCDC134. The ER signal sequence (characterized in (*17*)) and ER retrieval signal (QSEL; characterized here) are labeled. The position of the 3xHA sequence used to epitope tag CCDC134 in most of our experiments is noted with a red arrow.

(F) Fluorescence microscopy was used to assess the subcellular localization of 3xHA-CCDC134 (red) carrying its native QSEL ER retention sequence or a mutant QSSA sequence at the C-terminus after stable expression in *CCDC134*$^{-/-}$ cells. Giantin (green) was used to mark the Golgi. Wild-type CCDC134 was found in a reticular pattern throughout the cytoplasm, consistent with ER localization (top row). The CCDC134-QSSA mutant was mostly localized in the Golgi, a hallmark of secreted proteins (bottom row)(*48*). Scale bar: 10 µm.

(G) Abundance of 3xHA-CCDC134 carrying the native QSEL or mutant QSSA ER retention sequence retained in cells or secreted into media. The CCDC134 variants were stably expressed in *CCDC134*$^{-/-}$ cells under the control of a doxycycline-inducible promoter (**fig.S3G**). Cells and accompanying media were harvested for immunoblotting 24 hours after treatment with increasing concentrations of doxycycline (0, 2, 10, 50, 250 nM).

fig. S2

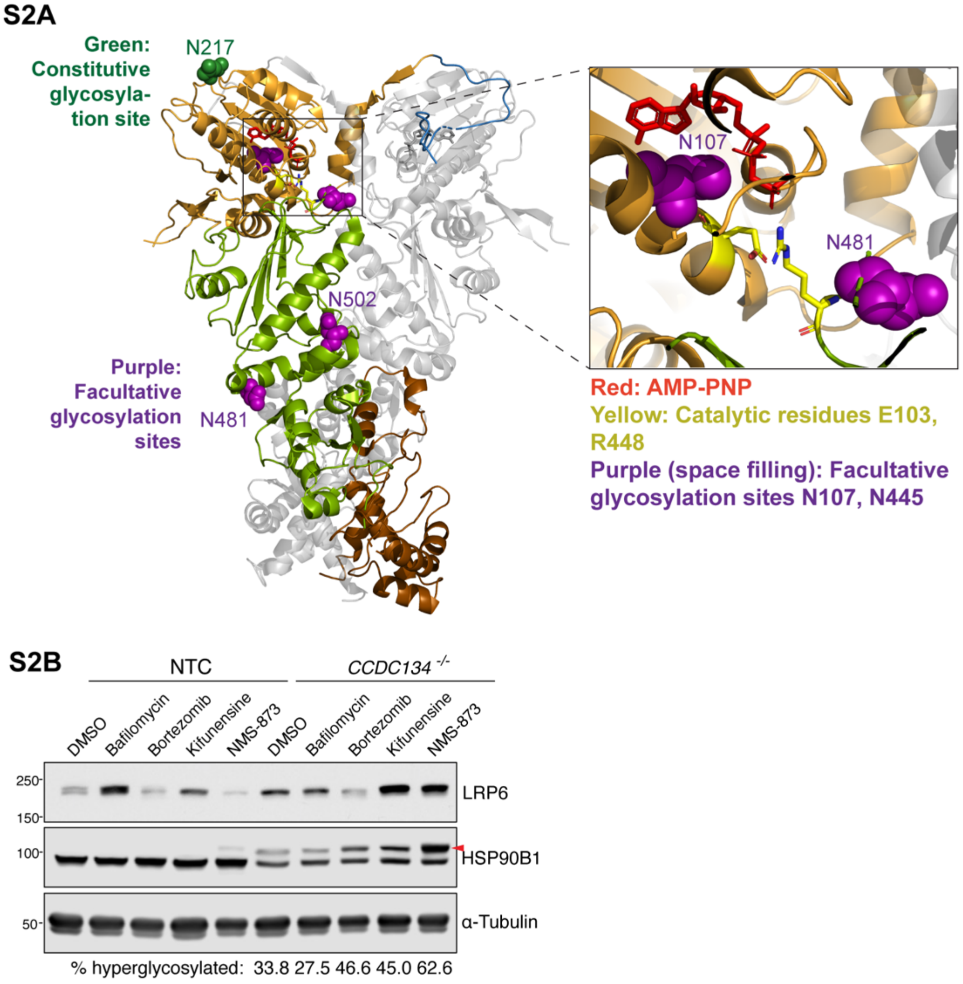

Supplementary Figure 2. HSP90B1 is hyperglycosylated at residues that would prevent its function or folding.

(A) Structure (PDB 5ULS) highlighting one protomer of a HSP90B1 dimer coloured to match the scheme used in **Fig.1D** (*49*). The N-terminal domain (NTD) binds to ATP, the Middle domain (MD) participates in ATP hydrolysis and client recognition, and the C-terminal domain (CTD) mediates dimerization. The ATP bound to the NTD and the asparagine side chains of the constitutive sequon (green) and the facultative sequons (purple) are shown in space filling representation. The inset shows proximity of two facultative sequons to the ATP binding site.

(B) Abundances and glycosylation status of HSP90B1 and LRP6 in lysates from clonally derived control (NTC) and *CCDC134*$^{-/-}$ RKO cells treated (18 hours) with small molecule inhibitors of (1) lysosomal acidification (50 nM Bafilomycin A1), (2) the proteasome (1 µM bortezomib), or (3) the ERAD pathway (5 µg/mL kifunensine or 5 µM NMS-873). As a control, media was also supplemented with an equivalent concentration of the solvent (DMSO) contributed by the inhibitor stock solutions. The hyperglycosylated HSP90B1 band is indicated with a red arrowhead. Numbers below the lanes show the percentage of total HSP90B1 that is hyperglycosylated; note the increase in extracts from bortezomib, kifunensine, and NMS-873 treated cells.

fig. S3

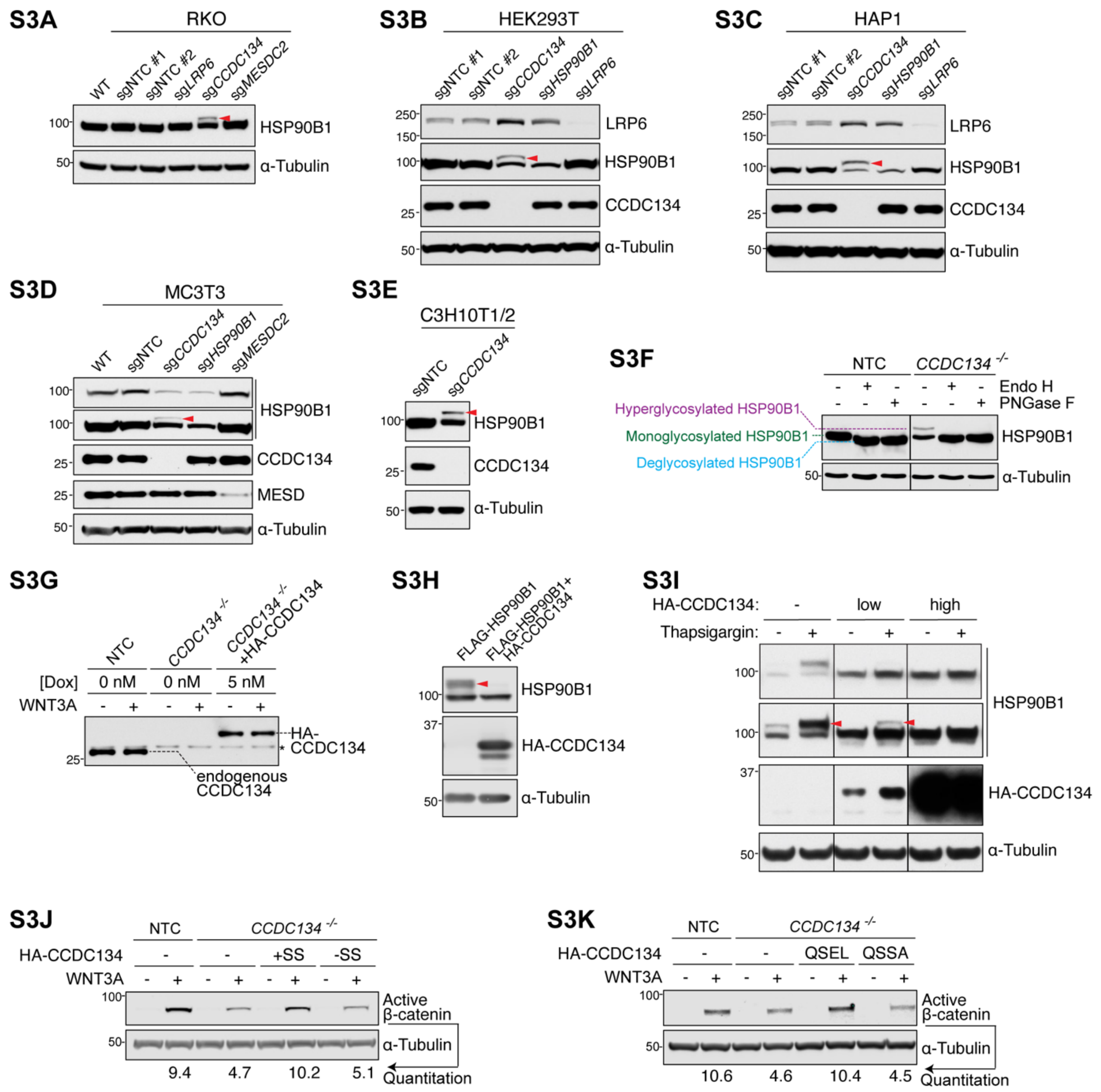

Supplementary Figure 3. CCDC134 suppresses the hyperglycosylation of HSP90B1 and promotes WNT signaling.

(A-E) LRP6 abundance and HSP90B1 hyperglycosylation (red arrowhead) in a panel of human (RKO, HEK293T, HAP1) and mouse (MC3T3, C3H10T1/2) cell lines expressing sgRNAs targeting the indicated genes (NTC=non-targeting control sgRNA).

(F) Glycosidase sensitivity (Endo H or PNGase F, see **Fig.1K**) of HSP90B1 in lysates of control (NTC) or *CCDC134*$^{-/-}$ cells. HSP90B1 is glycosylated at either a single constitutive site (monoglycosylated band) or at multiple facultative sites (hyperglycosylated bands) (see (*19*) and **Fig.1D**).

(G) Abundance of 3xHA-CCDC134 in cells stably expressing a doxycycline (Dox)-inducible transgene after treatment with 5 nM Dox was measured by immunoblotting with an anti-CCDC134 antibody. Abundance of endogenous CCDC134 in control (NTC) cells is shown as a comparison. These conditions were used throughout our experiments to achieve near-endogenous levels of 3xHA-CCDC134. ***** marks a nonspecific band.

(H) Impact of CCDC134 co-expression on HSP90B1 hyperglycosylation (red arrowhead) when the both proteins were transiently expressed in HEK293T cells.

(I) Effects of low and high 3xHA-CCDC134 expression (from a stably integrated, doxycycline-inducible transgene) on HSP90B1 hyperglycosylation (red arrowhead) in *CCDC134*$^{-/-}$ RKO cells treated with 0.1 uM thapsigargin for 24 hrs to induce ER stress. Low (near-endogenous, see **S3G**) and high CCDC134 expression was achieved by supplementing media with 5 nM or 25 nM doxycycline, respectively.

(J) Active β-catenin abundance (+/- WNT3A) in clonally derived control (NTC) and *CCDC134*$^{-/-}$ cell lines stably expressing near endogenous levels of 3xHA-CCDC134 (**S3G**) carrying an N-terminal ER signal sequence (+SS) or, as a control, lacking a signal sequence (-SS) to prevent ER targeting.

(K) Active β-catenin abundance (+/-WNT3A) in control or *CCDC134*$^{-/-}$ cells stably expressing the QSEL or QSSA variants of 3xHA-CCDC134 at near-endogenous levels (see **fig.S1E-S1G**).

In **S3J** and **S3K**, numbers below the lanes show the WNT3A-induced fold-change in active β-catenin abundance normalized to α-Tubulin abundance.

fig. S4

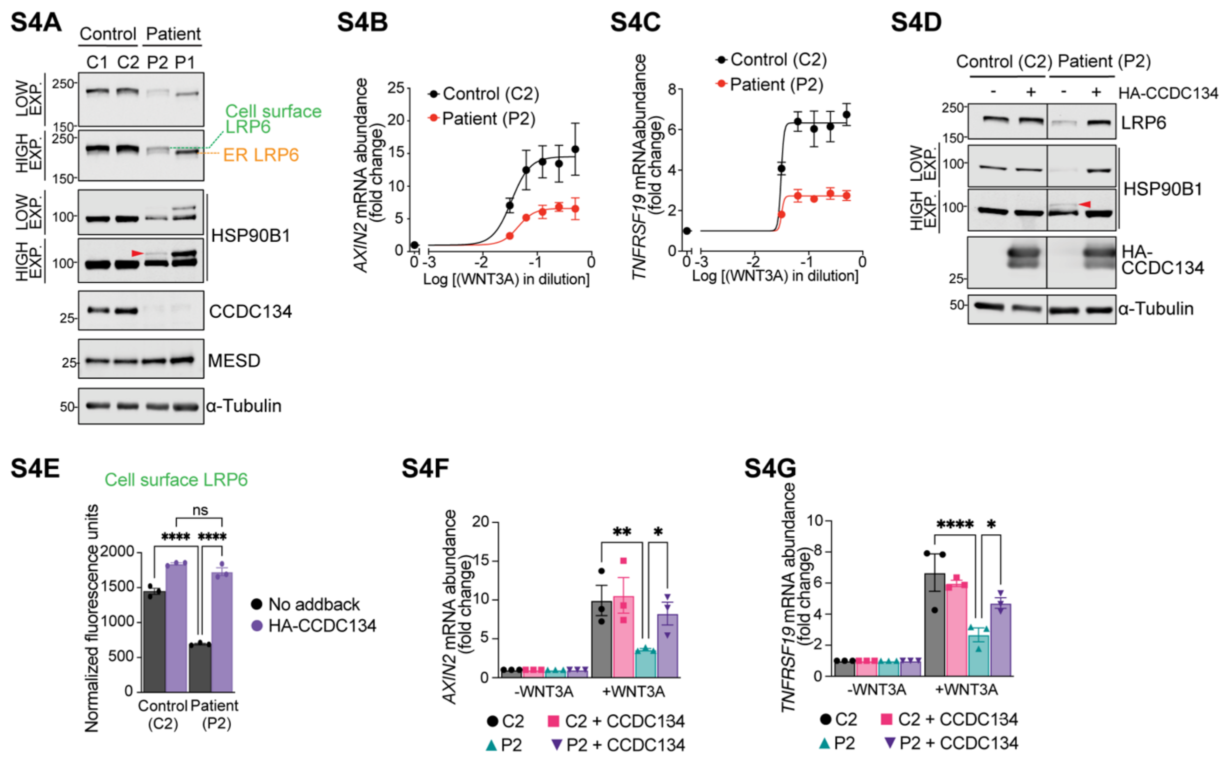

Supplementary Figure 4. HSP90B1 hyperglycosylation and LRP6 trafficking in human patients carrying *CCDC134* mutations.

(A) LRP6, HSP90B1 and CCDC134 abundances in lysates from primary fibroblasts isolated from two Osteogenesis Imperfecta (OI) patients carrying homozygous loss-of-function mutations in CCDC134 (c.2T>C) (P1, P2) and two age-matched healthy control individuals (C1 and C2, respectively)(*28*).

(B, C) WNT3A dose-response curves in OI patient and control fibroblasts. The abundances of *AXIN2* mRNA (**B**) or *TNFRSF19* mRNA (**C**), encoded by two different immediate-early WNT target genes, were measured by qRT-PCR as a metric of WNT signaling strength. Error bars show the mean +/- SEM from three independent experiments.

(D) Abundances of LRP6 and HSP90B1 in primary fibroblasts isolated from a *CCDC134*$^{-/-}$ patient with or without stable re-expression of 3xHA-CCDC134.

(E) Cell surface LRP6 abundance (mean +/- SEM from three independent experiments) measured by flow-cytometry in primary fibroblasts isolated from a *CCDC134*$^{-/-}$ patient with or without stable re-expression of 3xHA-CCDC134. Statistical significance was determined by two-way ANOVA with Tukey's multiple comparisons test; **** p<0.0001 (**K**).

(F,G) Expression of the WNT target genes *AXIN2* and *TNFRSF19* (mean +/- SEM of target gene mRNA normalized to *GAPDH* mRNA measured by qRT-PCR in three independent experiments) in primary patient fibroblasts stably expressing 3xHA-CCDC134 treated with 25% WNT3A conditioned media. Statistical significance was determined by two-way ANOVA with Dunnett's multiple comparisons test; * p<0.05, ** p<0.01, **** p<0.0001.

fig. S5

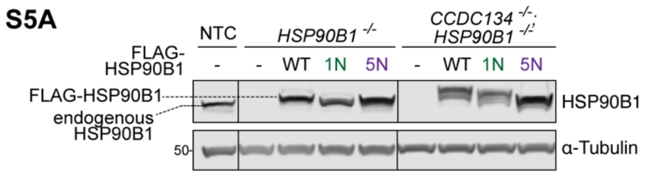

Supplementary Figure 5. Characterization of 1N and 5N variants of HSP90B1.

(A) Abundances of endogenous HSP90B1 in control (NTC) cells compared to stably integrated 3xFLAG-HSP90B1 variants in *HSP90B1*$^{-/-}$ and *CCDC134*$^{-/-}$;*HSP90B1*$^{-/-}$ cell lines (see **Fig.2A-2D**).

fig. S6

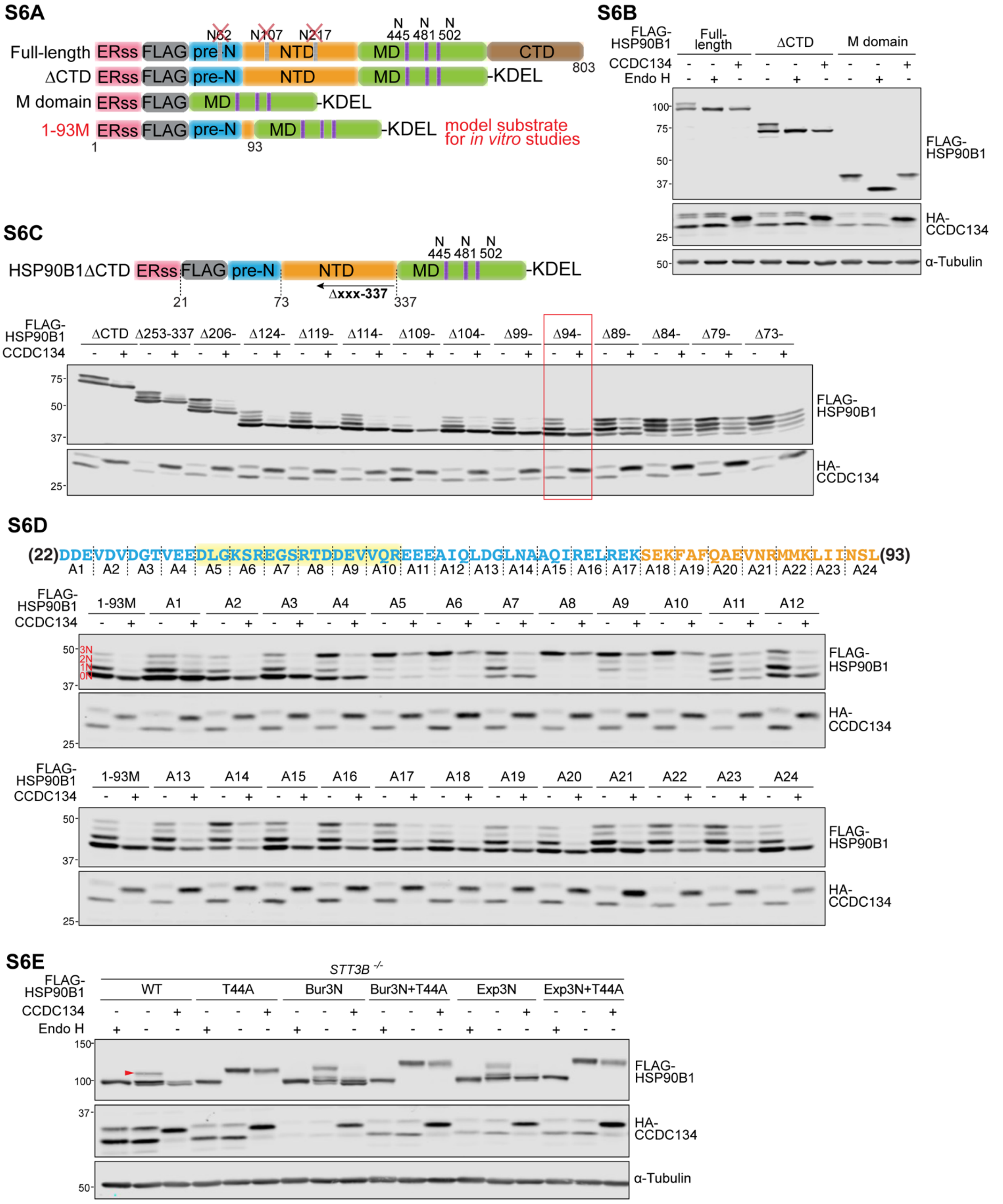
S6A
Full-length ERss FLAG pre-N NTD MD CTD
ΔCTD ERss FLAG pre-N NTD MD -KDEL
M domain ERss FLAG MD -KDEL
1-93M ERss FLAG pre-N MD -KDEL
model substrate for in vitro studies
S6B
FLAG-HSP90B1
CCDC134
Endo H
FLAG-HSP90B1
HA-CCDC134
α-Tubulin
S6C
HSP90B1ΔCTD ERss FLAG pre-N NTD MD -KDEL
Δxxx-337
FLAG-HSP90B1
HA-CCDC134
S6D
(22)DDEVDVDGTVEEDLGKSREGSRTDDEVVQREEEAIQLDGLNAAQIRELREKSEKFAFQAEVNRMMKLIINSL(93)
FLAG-HSP90B1
HA-CCDC134
S6E
STT3B -/-
FLAG-HSP90B1
CCDC134
Endo H
HA-CCDC134
α-Tubulin

Supplementary Figure 6. Mutagenesis identifies the sequence features of HSP90B1 that inhibit its own glycosylation.

(A) Variants of HSP90B1 used for cell-based and *in vitro* assays. Key features include the ERss, ER signal sequence; FLAG, 3xFLAG tag; pre-N, unstructured segment; NTD, N-terminal domain; MD, middle domain; CTD, C-terminal domain. N-glycosylation sites in the pre-N and NTD regions were eliminated to allow easy assessment of the glycan modification of the three sequons in the M domain by gel shifts (see **Fig.3B**).

(B) Glycosylation status of HSP90B1 variants shown in **S6A** was assessed using gel shifts and Endo H sensitivity after transient co-expression in HEK293T cells with WT CCDC134 (**+**) or a non-functional variant (**-**) lacking its ER signal sequence (see **Fig.S3J**).

(C) Glycosylation status of the indicated deletion mutants of FLAG-HSP90B1 lacking its CTD and containing only three sequons in the M domain (ΔCTD, see **S6A**). The deletion series starts at amino acid 337 and extends sequentially into the NTD and pre-N segments. The minimum construct that still retains regulation of glycosylation is boxed in red and named the 1-93M construct (**S6A**).

(D) Triplet alanine scanning mutagenesis was used to identify amino acid residues within the pre-N domain of HSP90B1 that regulate its own glycosylation. Sets of three consecutive residues (A1-A24, as shown in the pre-N sequence above the immunoblots) were mutated to Ala-Ala-Ala in the 1-93M variant of HSP90B1 (see **S6A**) and glycosylation tested by transiently transfecting the encoding constructs into HEK293T cells.

(E) Glycosylation status of full-length FLAG-HSP90B1 (**S6A**) carrying a T44A mutation in the "SRT" pseudosubstrate site identified in **Fig.3C**. Bur3N and Exp3N refer to variant proteins containing three additional artificial sequons predicted to be buried (Bur) or exposed (Exp) based on the PDB 5ULS HSP90B1 structure(*49*).

fig. S7

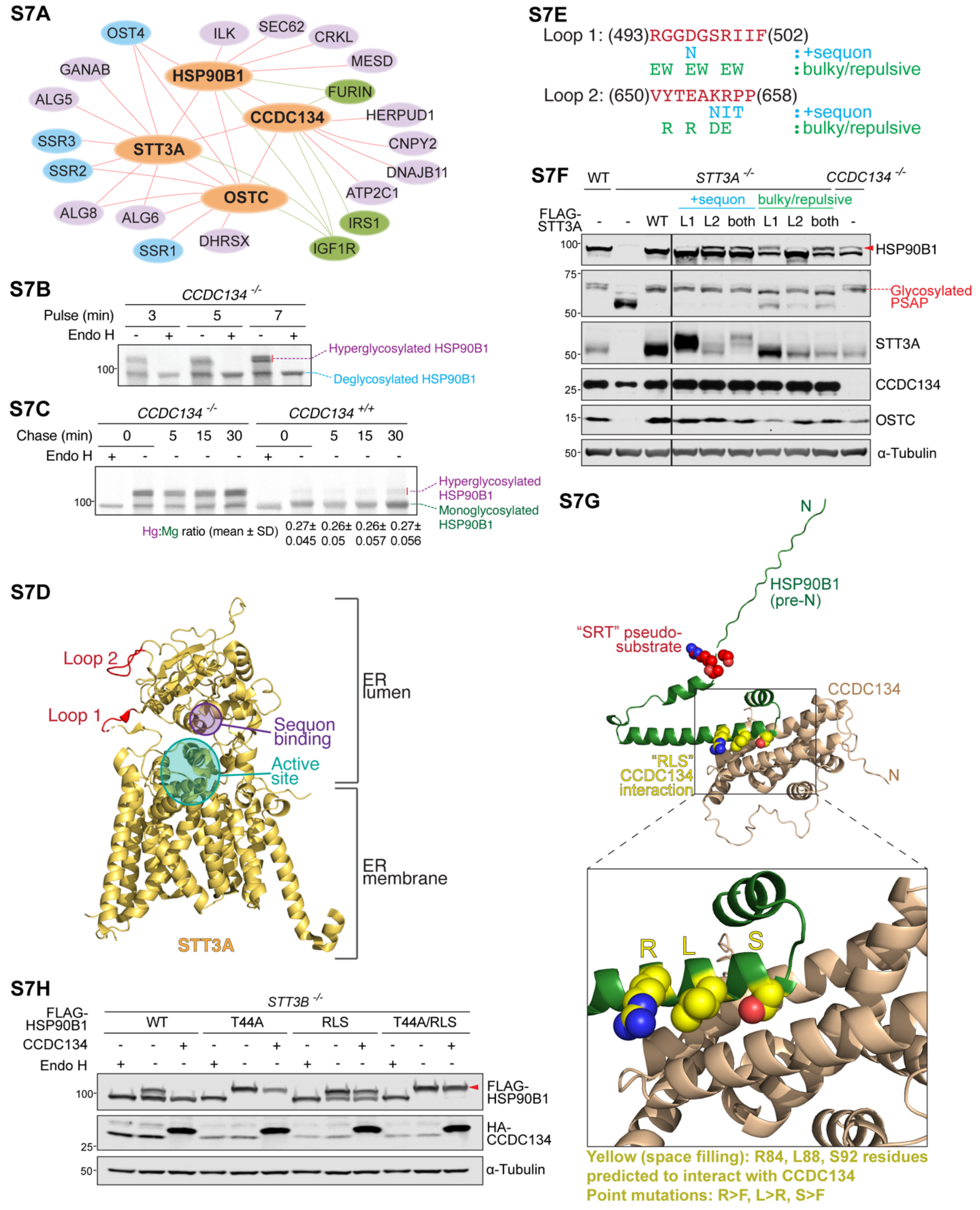

S7A
OST4
ILK
SEC62
CRKL
MESD
GANAB
HSP90B1
FURIN
ALG5
CCDC134
HERPUD1
SSR3
STT3A
CNPY2
SSR2
DNAJB11
ATP2C1
OSTC
ALG8
ALG6
IRS1
DHRSX
SSR1
IGF1R
S7B
CCDC134 -/-
Pulse (min) 3 5 7
Endo H - + - + - +
100
Hyperglycosylated HSP90B1
Deglycosylated HSP90B1
S7C
CCDC134 -/-
CCDC134 +/+
Chase (min) 0 5 15 30 0 5 15 30
Endo H + - - - - + - - - -
100
Hyperglycosylated HSP90B1
Monoglycosylated HSP90B1
Hg:Mg ratio (mean ± SD)
0.27± 0.045
0.26± 0.05
0.26± 0.057
0.27± 0.056
S7D
Loop 2
Loop 1
Sequon binding
Active site
ER lumen
ER membrane
STT3A
S7E
Loop 1: (493)RGGDGSRIIF(502)
N :+sequon
EW EW EW :bulky/repulsive
Loop 2: (650)VYTEAKRPP(658)
NIT :+sequon
R R DE :bulky/repulsive
S7F
WT
STT3A -/-
CCDC134 -/-
+sequon
bulky/repulsive
FLAG-STT3A - - WT L1 L2 both L1 L2 both -
100
HSP90B1
75
50
Glycosylated PSAP
50
STT3A
25
CCDC134
15
OSTC
50
α-Tubulin
S7G
N
HSP90B1 (pre-N)
"SRT" pseudo-substrate
CCDC134
"RLS" CCDC134 interaction
N
R
L
S
Yellow (space filling): R84, L88, S92 residues predicted to interact with CCDC134
Point mutations: R>F, L>R, S>F
S7H
STT3B -/-
FLAG-HSP90B1 WT T44A RLS T44A/RLS
CCDC134 - - + - - + - - + - - +
Endo H + - - + - - + - - + - -
100
FLAG-HSP90B1
25
HA-CCDC134
50
α-Tubulin

Supplementary Figure 7. Separation-of-function mutations show that CCDC134 is recruited to the translocon through interactions with both STT3A and HSP90B1.

(A) DepMap cluster centered on CCDC134, STT3A, HSP90B1, and OSTC, expanded (compared to **Fig.1A**) to include the top 10 co-essential genes of each input node (*16*). Genes encoding components of the secretory ER translocon and genes regulating IGF1R receptor biogenesis and signaling are colored blue and green, respectively.

(B) Pulse-labeling of HSP90B1 in clonal *HSP90B1*$^{-/-}$;*CCDC134*$^{-/-}$ double knock-out cells stably expressing 3xFLAG-HSP90B1. Culture media was supplemented with ^{35}S-Methionine and ^{35}S-Cysteine for 3, 5 or 7 minutes, followed by lysis and immunoprecipitation of HSP90B1 on anti-FLAG beads. The Endo H-treated sample is equivalent to 33% of the undigested sample. This data supports a cotranslational mode of hyperglycosylation because the hyperglycosylated HSP90B1 band was detected at the earliest time points after the addition of ^{35}S-Methionine, concomitant with the detection of the monoglycosylated band.

(C) Pulse labeling (5 min) of HSP90B1 with ^{35}S-Methionine/^{35}S-Cysteine in *HSP90B1*$^{-/-}$ or *HSP90B1*$^{-/-}$;*CCDC134*$^{-/-}$ cells stably expressing 3xFLAG-HSP90B1 was followed by a chase in unlabelled media for 5, 15 or 30 minutes. The ratio of the hyperglycosylated (Hg) to monoglycosylated (Mg) HSP90B1 band intensity is indicated from three independent experiments. The lack of any evidence of label transfer from monoglycosylated to hyperglycosylated HSP90B1 supports co-translational (rather than post-translational) N-glycosylation.

(D,E) Structure of STT3A (PDB 6S7O (*7*)) highlighting the two ER luminal loops (colored red) that were tested as potential interaction motifs for CCDC134. The active site (teal) and sequon binding site (purple) are labeled in relation to the two loops. **(E)** shows the loop sequences along (in red text) with mutations designed to impair the interaction with CCDC134. Loops were altered either to introduce ectopic N-glycosylation sites (blue) or to reverse the charge and introduce steric bulk (green).

(F) Glycosylation status and abundances of endogenous HSP90B1 in *CCDC134*$^{-/-}$ cells (rightmost lane) or *STT3A*$^{-/-}$ cells stably expressing wild-type FLAG-STT3A or variants that have mutations (listed in **S7E**) in Loop 1 (L1), Loop 2 (L2) or both loops ("both"). Red arrowhead points to the hyperglycosylated HSP90B1 population. Glycosylation of the canonical OST-A substrate PSAP (which is independent of CCDC134, **Fig.3E**) was used to assess the function of each STT3A variant. Cells expressing STT3A variants with mutations in these loops show an increase in HSP90B1 hyperglycosylation (similar to what is seen in *CCDC134*$^{-/-}$ cells) but minimal defect in PSAP N-glycosylation. These results indicate that CCDC134 function is impaired by L1 and L2 mutations in STT3A.

(G) AlphaFold3 model of the complex between CCDC134 and the pre-N segment of HSP90B1. Side chain of residues that comprise the SRT pseudosubstrate motif and the RLS motif at the interface with CCDC134 are shown in space-filling representation. Inset shows a close-up of the predicted pre-N-CCDC134 interaction site.

(H) Glycosylation status of full-length HSP90B1 carrying mutations in the SRT pseudosubstrate site (T44A, **Fig.3C**), the RLS site (RLS-->FRF) (see **S7G**) or both sites was assessed using gel shifts and Endo H sensitivity after transient co-expression in HEK293T cells with WT CCDC134 (**+**) or a non-functional variant (**-**) lacking its ER signal sequence (see **Fig.S3J**). Red arrowhead points to the hyperglycosylated HSP90B1 population.

fig. S8

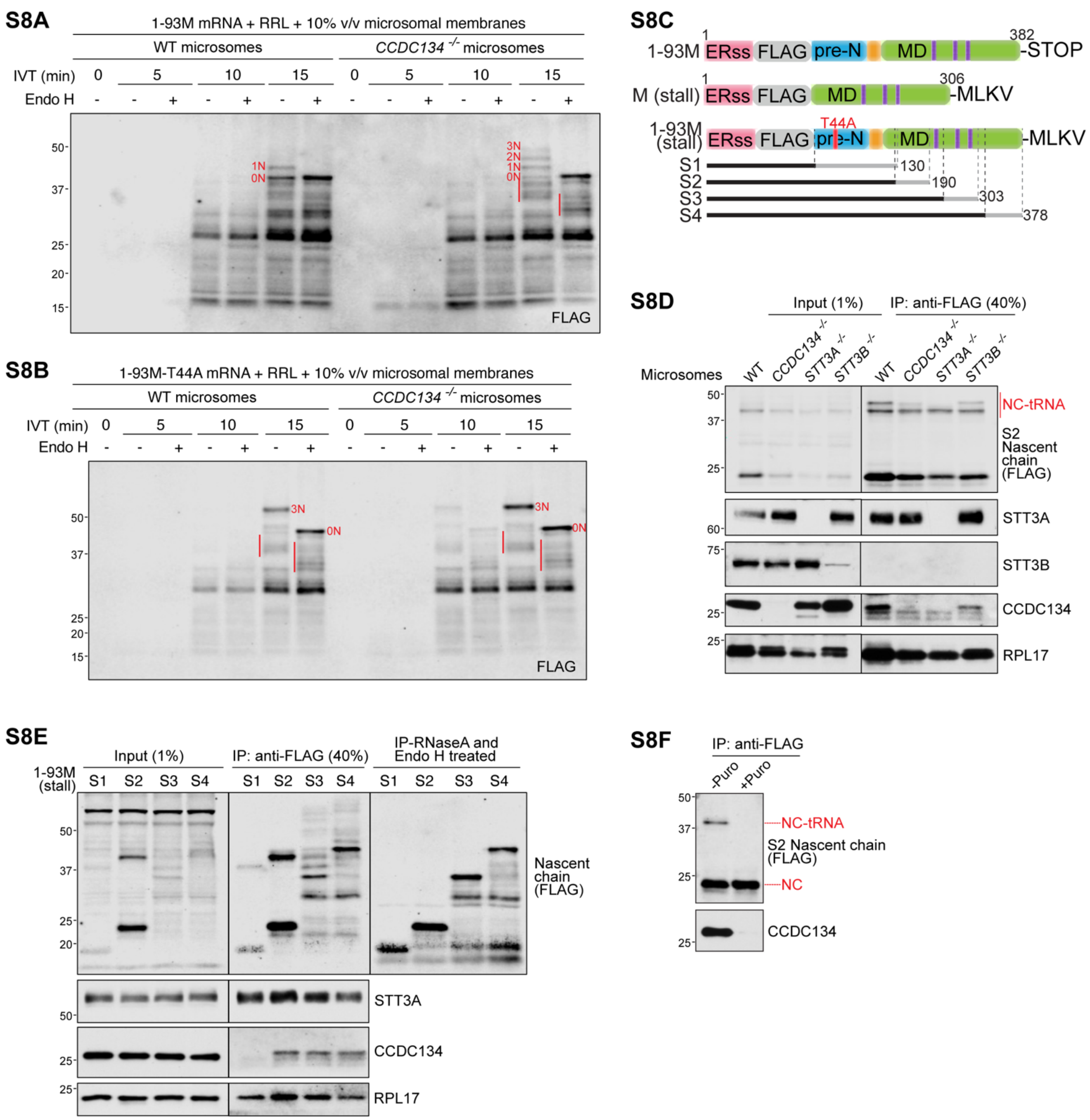

Supplementary Figure 8. Co-translational association of CCDC134 with the secretory ER translocon.

(A, B) Translation time course of 1-93M (**A**) or 1-93M carrying the T44A mutation in the "SRT" pseudosubstrate site (**B**) in rabbit reticulocyte lysate (RRL) supplemented with rough microsomes isolated from wild-type or *CCDC134*$^{-/-}$ HEK293T cells. The sizes and Endo H sensitivities of the translation products were analyzed after anti-FLAG immunoprecipitation. N-glycosylated translational intermediates (highlighted by a red line) were identified by their size (shorter than the full-length protein seen at 15 min) and sensitivity to Endo H. Co-translational glycosylation is supported by the detection of N-glycosylated nascent peptides that were shorter than the full-length protein.

(C) Constructs used for *in vitro* translation experiments. For the four variants of the 1-93M (stall) constructs, named S1-S4, the dark gray indicates the region predicted to be fully in the ER lumen and light gray indicates the region predicted to be in the translocon and ribosome exit tunnel.
(D) Association of endogenous CCDC134 with a stalled S2 nascent chain translated in the presence of microsomes isolated from wild-type (WT), *CCDC134*$^{-/-}$, *STT3A*$^{-/-}$, or *STT3B*$^{-/-}$ HEK293T cells. The stalled nascent chain was immunoprecipitated (IP) using anti-FLAG beads and association with STT3A, CCDC134, and the ribosome (RPL17) assessed by immunoblotting. NC-tRNA: nascent chain-tRNA conjugates.
(E) Association of endogenous CCDC134 with the S1-S4 truncation variants of 1-93M (diagrammed in **S8C**) lacking a STOP codon translated in the presence of rough microsomes generated from wild-type HEK293T cells. To clearly visualize the sizes of the truncation variants, each sample was also treated with RNase A to release tRNA and Endo H to collapse glycoforms (top right immunoblot).
(F) Association of CCDC134 with a stalled S2 nascent chain in the presence or absence of puromycin. Puromycin releases the nascent chain from the P-site tRNA in the ribosome, as seen by the loss of the band corresponding to the nascent chain (NC)-tRNA conjugate.

fig. S9

S9A

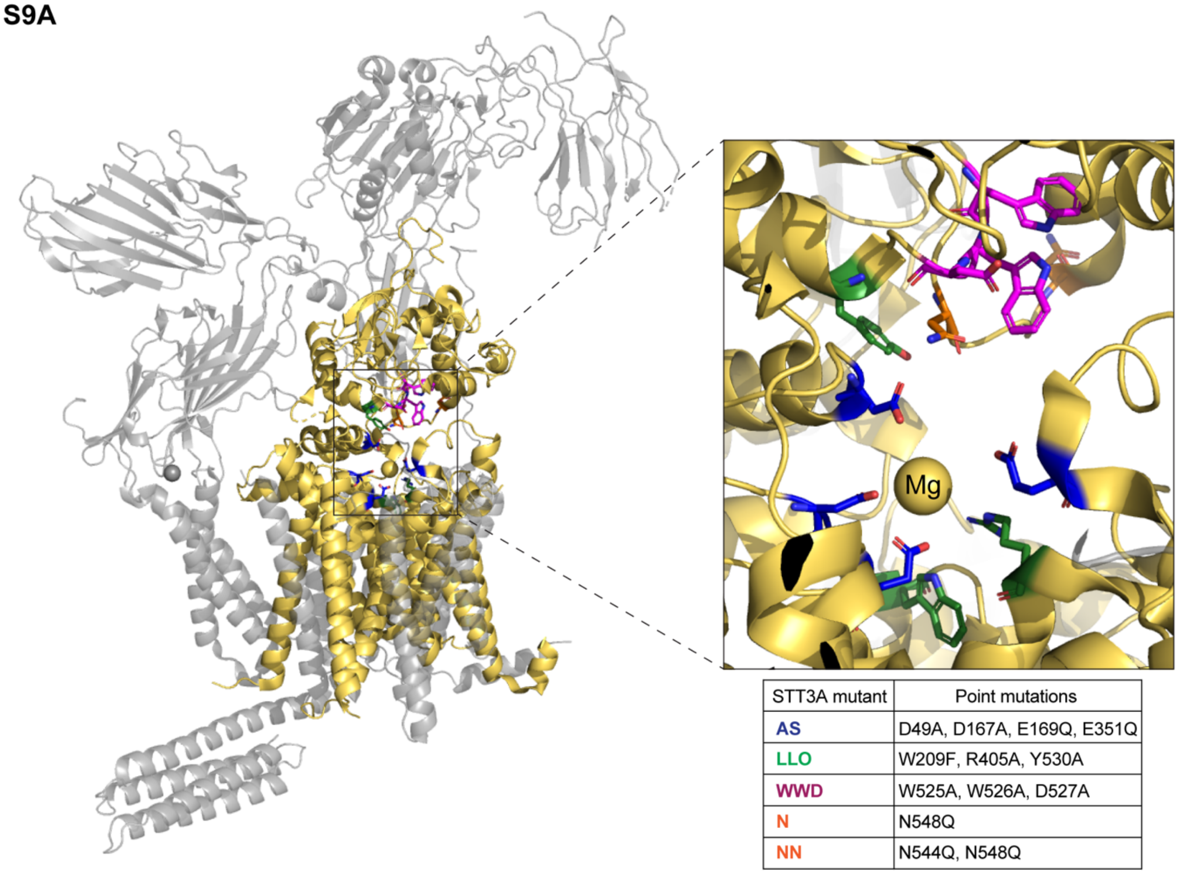

STT3A mutant	Point mutations
AS	D49A, D167A, E169Q, E351Q
LLO	W209F, R405A, Y530A
WWD	W525A, W526A, D527A
N	N548Q
NN	N544Q, N548Q

S9B

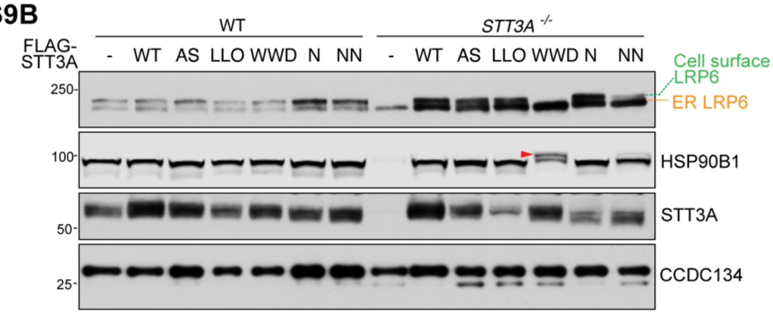

Supplementary Figure 9. Catalytic activity of STT3A is not required to regulate HSP90B1 hyperglycosylation.

(A) Cryo-EM structure of the OST-A complex (PDB 6S7O (*7*)) with STT3A highlighted in yellow. Residues that were mutated to generate the STT3A variants tested in **Fig.4 and fig.S9B** are shown on the structure and listed in the table below. Variants carry mutations in residues involved in active site chemistry (AS, blue), lipid-linked oligosaccharide binding (LLO, green), sequon binding (WWD, magenta) or N-glycosylation of STT3A itself (N and NN, orange).

(B) Glycosylation status and abundances of LRP6, HSP90B1, STT3A and CCDC134 in wild-type (WT) or *STT3A*$^{-/-}$ cells stably expressing FLAG-STT3A variants carrying mutations in various sites (listed in **S9A**) involved in catalytic transfer of the glycan from the lipid-linked oligosaccharide to the asparagine in sequons. See **Fig.4A and 4B**.

fig. S10

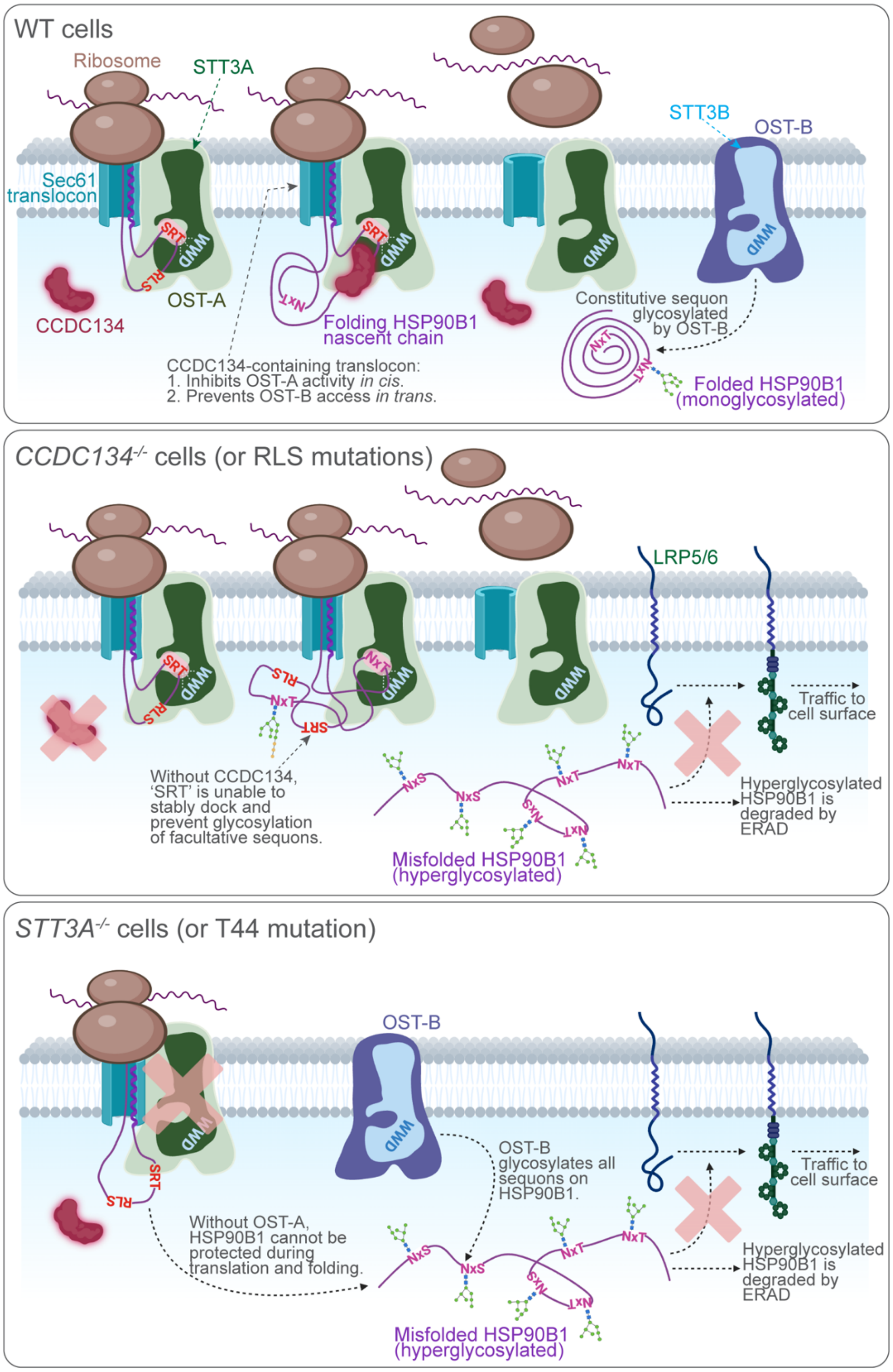

Supplementary Figure 10. Models for HSP90B1 N-glycosylation in wild-type, *CCDC134*$^{-/-}$ and *STT3A*$^{-/-}$ cells.

In WT cells (top), CCDC134 is recruited to secretory translocons during translation of HSP90B1, resulting in 1) inhibition of STT3A activity toward HSP90B1 sequons and 2) prevention of STT3B access to the same sequons during HSP90B1 folding. The distinct, mutationally separable roles of the inhibitory activity of the HSP90B1 pre-N segment and the recruitment of CCDC134 are supported by the

separation-of-function mutations detailed in **fig.S7**. In *CCDC134*$^{-/-}$ cells (middle), the SRT pseudosubstrate site does not stably dock in the sequon-binding site of STT3A, leading to an increase in HSP90N1 hyperglycosylation. Loss of STT3A (bottom) completely disrupts the translocon-proximal protective scaffold, allowing OST-B to fully N-glycosylate all available sequons in HSP90B1, eventually leading to its degradation by ERAD.

fig. S11

S11A

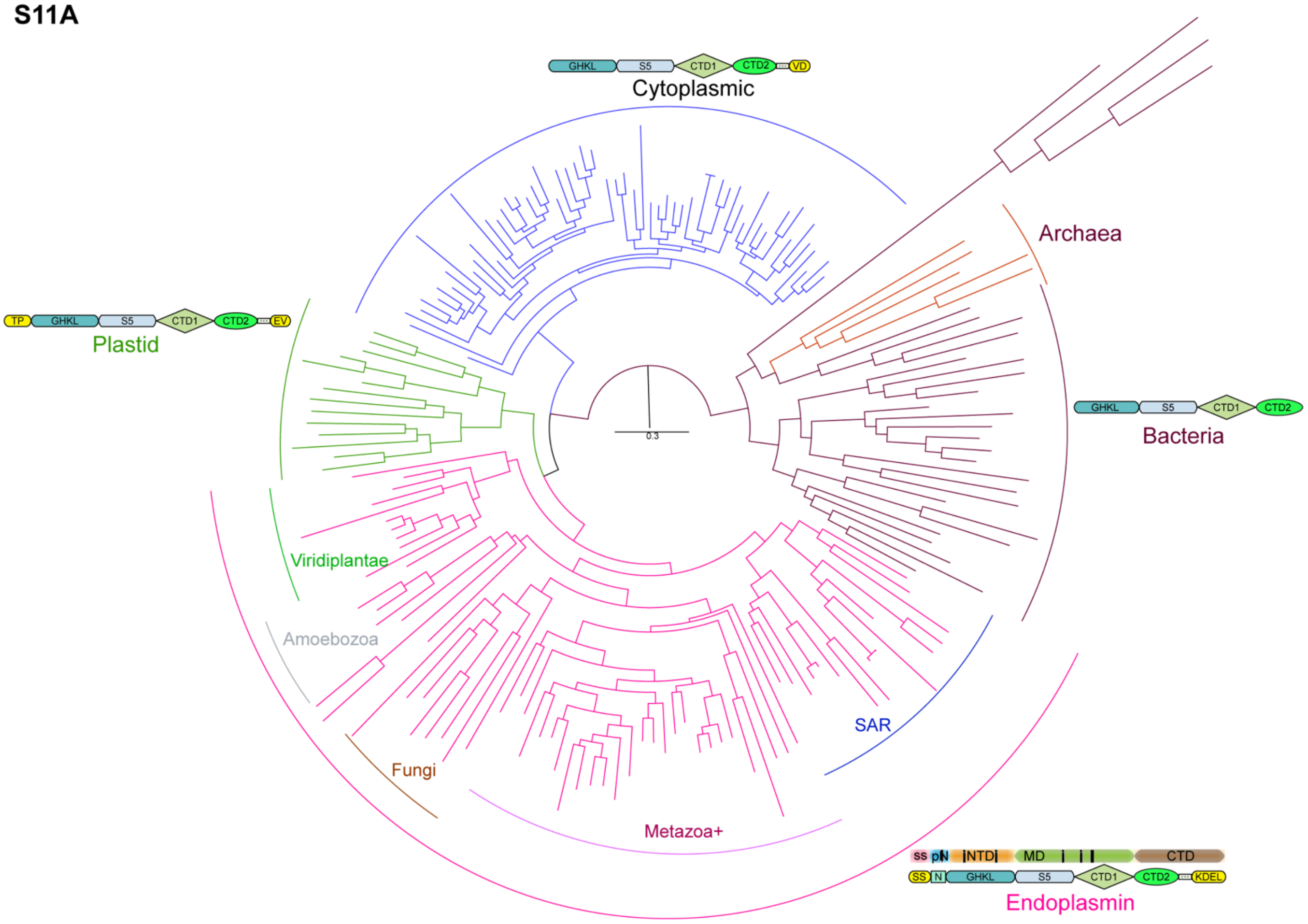

S11B

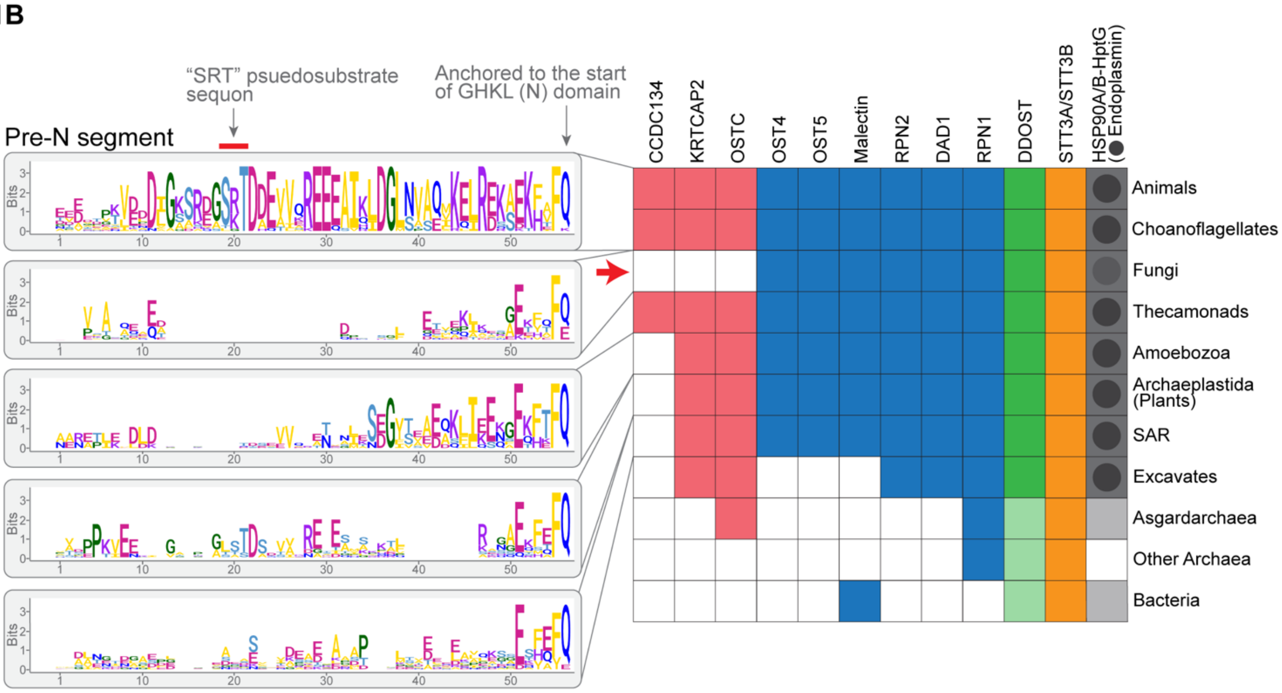

Supplementary Figure 11. Evolutionary analysis of HSP90B1, its pre-N segment and CCDC134.

(A) HSP90B1 is a member of the HSP90 family of dimeric ATP-dependent chaperones that are conserved across the tree of life. Within the HSP90 family, HSP90B1 belongs to the endoplasmin clade characterized by three unique sequence features: a signal sequence for targeting to the ER, a KDEL or equivalent ER-retention sequence, and the unstructured pre-N segment that is a focus of this work. In this tree, the 4 major clades (prokaryotic, eukaryotic cytoplasmic, eukaryotic plastid, and eukaryotic endoplasmin) are colored distinctly. For each clade the formal domain architecture is indicated. For the endoplasmins, the HSP90B1 domain diagram used throughout this manuscript is juxtaposed for comparison and glycosylation sites are marked as black lines. Within the endoplasmin clade (which includes HSP90B1), the subclades corresponding to major eukaryotic lineages are marked separately. Metazoa+ indicates the Metazoa+ sister lineages choanoflagellates and Filasterians. Other annotated features include the S5 region, which supplies the lysine that stabilizes the hypercharged state during hydrolysis; CTD1 and CTD2, two domains which together are called the C-terminal domain; GHKL, ancient ATP binding domain found in Gyrase, Hsp90, histidine Kinases and MutL; TP, transit peptide in plastid HSP90; EV/VD/KDEL, terminal motifs.

(B) Conservation of CCDC134, non-catalytic OST subunits, STT3A/B, HSP90 and endoplasmin (which includes HSP90B1 in mammals) across evolution. Shading indicates the presence of the protein in a specific sub-clade, and a black dot in the HSP90 column indicates the presence of a member of the endoplasmins. The conservation pattern of the pre-N segment in each sub-clade is shown as a sequence logo to the left. The logos are anchored to the start of the HSP90 NTD (the ATP-binding GHKL domain, see **S11A**) and the residues are scaled according to the conservation bit score. The concomitant loss of CCDC134 and OST-A-specific subunits OSTC and KRTCAP2 (that anchor it to the SEC61 translocon) in fungi is highlighted with a red arrow. The sequence logos show that pre-N domain conservation is correlated with the presence of CCDC134, with both being lost together in fungi.

fig. S12

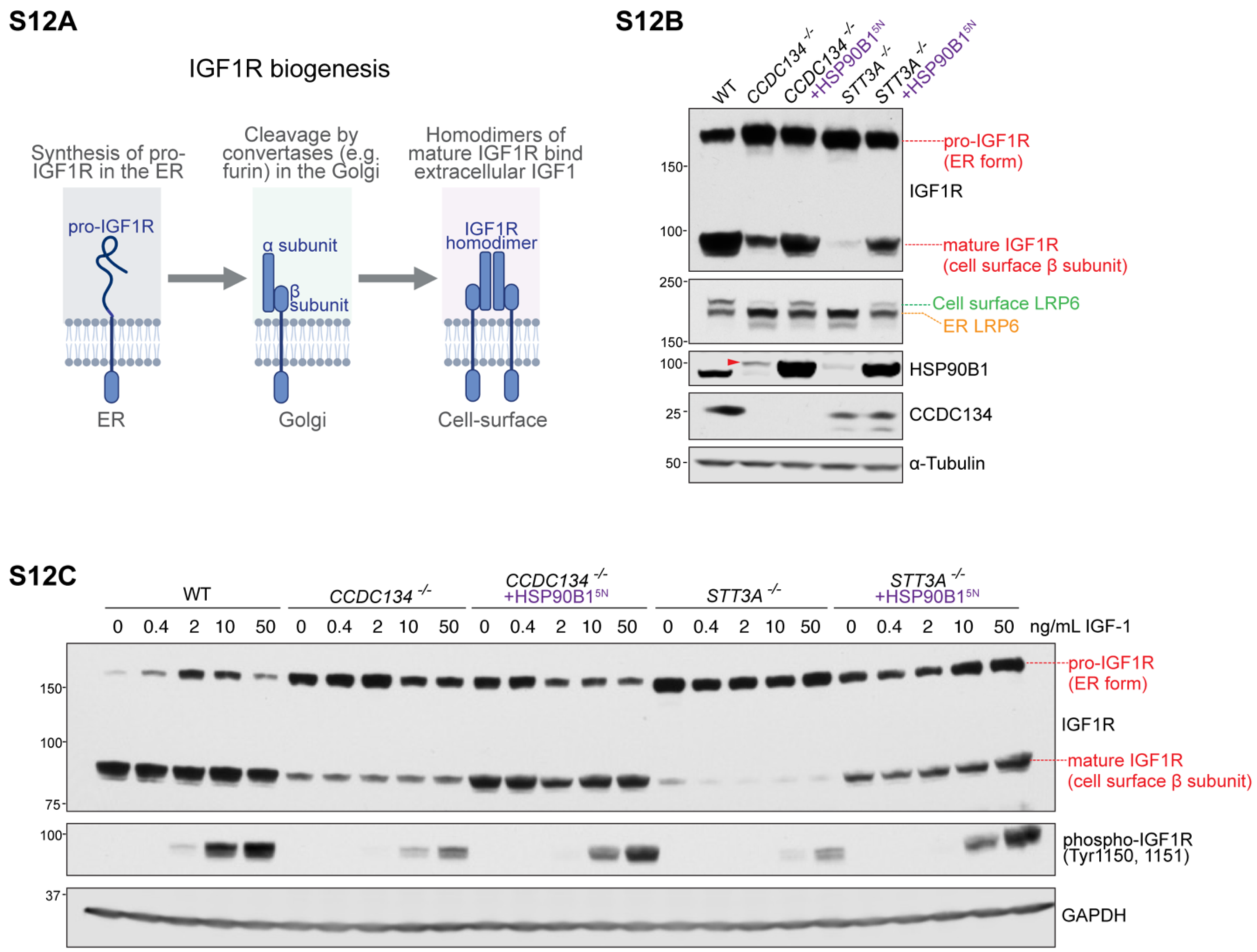

Supplementary Figure 12. Cell surface abundance of IGF1R is regulated by CCDC134 and HSP90B1 hyperglycosylation.

(A) IGF1R is synthesized as a longer precursor polypeptide in the ER (pro-IGF1R) that undergoes cleavage by proprotein convertases in the Golgi to produce a mature, disulfide-linked tetramer (composed of two α and two β subunits) found on the cell surface (*46*). Abundance of the shorter β subunit is commonly used as a measure of cell-surface IGF1R.

(B) Abundances of LRP6 and IGF1R in cells of the indicated genotypes. The HSP90B1^{5N} variant (**Fig.1D**) was stably expressed in *CCDC134*$^{-/-}$ and *STT3A*$^{-/-}$ cells to test if it can bypass the requirement of CCDC134 or STT3A for IGF1R cell surface expression (as it can for LRP6 cell surface expression, **Fig.2A and 2C**). Red arrowhead points to the population of HSP90B1 that is hyperglycosylated.

(C) Tyrosine phosphorylation of IGF1R was used as a measure of cell surface receptor activation in response to increasing concentrations of extracellular IGF1 supplied to cells of the indicated genotypes.

REFERENCES

48. I. Raykhel, H. Alanen, K. Salo, J. Jurvansuu, V. D. Nguyen, M. Latva-Ranta, L. Ruddock, A molecular specificity code for the three mammalian KDEL receptors. *J. Cell Biol.* **179**, 1193–1204 (2007).

49. J. D. Huck, N. L. Que, F. Hong, Z. Li, D. T. Gewirth, Structural and Functional Analysis of GRP94 in the Closed State Reveals an Essential Role for the Pre-N Domain and a Potential Client-Binding Site. *Cell Rep.* **20**, 2800–2809 (2017).

50. E. Campeau, V. E. Ruhl, F. Rodier, C. L. Smith, B. L. Rahmberg, J. O. Fuss, J. Campisi, P. Yaswen, P. K. Cooper, P. D. Kaufman, A versatile viral system for expression and depletion of proteins in mammalian cells. *PLoS One* **4**, e6529 (2009).

51. C. D. Marceau, A. S. Puschnik, K. Majzoub, Y. S. Ooi, S. M. Brewer, G. Fuchs, K. Swaminathan, M. A. Mata, J. E. Elias, P. Sarnow, J. E. Carette, Genetic dissection of Flaviviridae host factors through genome-scale CRISPR screens. *Nature* **535**, 159–163 (2016).

52. G. V. Pusapati, J. H. Kong, B. B. Patel, A. Krishnan, A. Sagner, M. Kinnebrew, J. Briscoe, L. Aravind, R. Rohatgi, CRISPR Screens Uncover Genes that Regulate Target Cell Sensitivity to the Morphogen Sonic Hedgehog. *Dev. Cell* **44**, 113–129.e8 (2018).

53. J. G. Doench, N. Fusi, M. Sullender, M. Hegde, E. W. Vaimberg, K. F. Donovan, I. Smith, Z. Tothova, C. Wilen, R. Orchard, H. W. Virgin, J. Listgarten, D. E. Root, Optimized sgRNA design to maximize activity and minimize off-target effects of CRISPR-Cas9. *Nat. Biotechnol.* **34**, 184–191 (2016).

54. J. Joung, S. Konermann, J. S. Gootenberg, O. O. Abudayyeh, R. J. Platt, M. D. Brigham, N. E. Sanjana, F. Zhang, Genome-scale CRISPR-Cas9 knockout and transcriptional activation screening. *Nat. Protoc.* **12**, 828–863 (2017).

55. W. Li, H. Xu, T. Xiao, L. Cong, M. I. Love, F. Zhang, R. A. Irizarry, J. S. Liu, M. Brown, X. S. Liu, MAGeCK enables robust identification of essential genes from genome-scale CRISPR/Cas9 knockout screens. *Genome Biol.* **15**, 554 (2014).

56. V. S. W. Li, S. S. Ng, P. J. Boersema, T. Y. Low, W. R. Karthaus, J. P. Gerlach, S. Mohammed, A. J. R. Heck, M. M. Maurice, T. Mahmoudi, H. Clevers, Wnt signaling through inhibition of β-catenin degradation in an intact Axin1 complex. *Cell* **149**, 1245–1256 (2012).

57. N. E. Sanjana, O. Shalem, F. Zhang, Improved vectors and genome-wide libraries for CRISPR screening. *Nat. Methods* **11**, 783–784 (2014).

58. A. Bermudez, S. J. Pitteri, Enrichment of Intact Glycopeptides Using Strong Anion Exchange and Electrostatic Repulsion Hydrophilic Interaction Chromatography. *Methods Mol. Biol.* **2271**, 107–120 (2021).

59. E. Shishkova, A. S. Hebert, M. S. Westphall, J. J. Coon, Ultra-High Pressure (>30,000 psi) Packing of Capillary Columns Enhancing Depth of Shotgun Proteomic Analyses. *Anal. Chem.* **90**, 11503–11508 (2018).

60. N. M. Riley, S. A. Malaker, M. D. Driessen, C. R. Bertozzi, Optimal Dissociation Methods Differ for N- and O-Glycopeptides. *J. Proteome Res.* **19**, 3286–3301 (2020).

61. J. Cox, M. Mann, MaxQuant enables high peptide identification rates, individualized

p.p.b.-range mass accuracies and proteome-wide protein quantification. *Nat. Biotechnol.* **26**, 1367–1372 (2008).

62. D. R. Brademan, I. J. Miller, N. W. Kwiecien, D. J. Pagliarini, M. S. Westphall, J. J. Coon, E. Shishkova, Argonaut: A Web Platform for Collaborative Multi-omic Data Visualization and Exploration. *Patterns (N Y)* **1** (2020).

63. D. A. Polasky, F. Yu, G. C. Teo, A. I. Nesvizhskii, Fast and comprehensive N- and O-glycoproteomics analysis with MSFragger-Glyco. *Nat. Methods* **17**, 1125–1132 (2020).

64. P. Walter, G. Blobel, Preparation of microsomal membranes for cotranslational protein translocation. *Methods Enzymol.* **96**, 84–93 (1983).
